# Comparison of large networks with sub-sampling strategies

**DOI:** 10.1038/srep28955

**Published:** 2016-07-06

**Authors:** Waqar Ali, Anatol E. Wegner, Robert E. Gaunt, Charlotte M. Deane, Gesine Reinert

**Affiliations:** 1 Department of Statistics, University of Oxford, 24-29 St: Giles’, Oxford OX1 3LB, UK

## Abstract

Networks are routinely used to represent large data sets, making the comparison of networks a tantalizing research question in many areas. Techniques for such analysis vary from simply comparing network summary statistics to sophisticated but computationally expensive alignment-based approaches. Most existing methods either do not generalize well to different types of networks or do not provide a quantitative similarity score between networks. In contrast, alignment-free topology based network similarity scores empower us to analyse large sets of networks containing different types and sizes of data. Netdis is such a score that defines network similarity through the counts of small sub-graphs in the local neighbourhood of all nodes. Here, we introduce a sub-sampling procedure based on neighbourhoods which links naturally with the framework of network comparisons through local neighbourhood comparisons. Our theoretical arguments justify basing the Netdis statistic on a sample of similar-sized neighbourhoods. Our tests on empirical and synthetic datasets indicate that often only 10% of the neighbourhoods of a network suffice for optimal performance, leading to a drastic reduction in computational requirements. The sampling procedure is applicable even when only a small sample of the network is known, and thus provides a novel tool for network comparison of very large and potentially incomplete datasets.

Data in many areas of science is now routinely represented in the form of networks. A fundamental task often required is to compare two datasets (networks) to assess the level of similarity between them. In the context of social networks, one might be interested in the structural differences between Facebook friendship networks and LinkedIn contact networks of individuals to test hypotheses about how relationships develop across different social spaces. In the biological sciences, networks often represent either direct or indirect molecular interactions and an active research area is to assess the level of conservation of interaction patterns across species. Many techniques for network comparison have been proposed in the literature, depending upon the targeted application domain and the size of datasets expected to be tackled.

The simplest and most tractable methods compare at the level of the entire network using summary statistics that describe global properties^1^. These global statistics offer a coarse-grained view of the topology of the network and are frequently not sensitive enough to distinguish between networks emerging from distinct underlying processes or models. At the opposite end of the spectrum are alignment based methods, where an optimal mapping between the nodes of two or more networks are sought, maximizing the agreement between node properties and their interaction behaviour. These methods have largely been applied in the biological network domain^2^,^3^,^4^, but are highly sensitive to noise and incompleteness in the data. Although the computational and error tolerance issues have been alleviated to some extent in more recent methods^5^,^6^,^7^, the concept is in general not applicable to other types of networks (for example random graph models).

In contrast, alignment-free methods, such as Netdis^8^, define similarity between networks based purely on their fine-grained topology. Netdis relies on the content of small subgraphs in the immediate neighbourhood (or ego-network) of each node in the original network (see Methods). The idea of using the subgraph content to build a distance between networks arises because motifs and modules have long been identified as important components of networks from multiple domains^9^,^10^,^11^. In particular, modules have been conjectured to play an important role in biological network evolution^12^,^13^; see Cootes *et al*.^14^ and Przujl^15^ and for previous explorations of biological network comparison based on subgraph counts. Netdis compares two networks through the collated subgraph counts in the ensembles of ego-networks from each larger network. Through the use of multiple samples the method should possess better statistical properties than simply comparing the counts of subgraphs in the original networks or using coarse global statistics. Netdis differs from standard subgraph count approaches in two key aspects: it introduces the ensemble view (via ego-networks) and applies a standardization which controls for background noise.

While Netdis offers substantial improvements in computational time over alignment-based comparison methods, the process of counting subgraphs in each ego-network represents a bottle-neck potentially restricting the application of the method when dealing with very large graphs (e.g 100,000 nodes). In this paper, we address this issue by introducing ego-network sub-sampling in the Netdis framework. Subsampling or bootstrapping on networks has been given little attention in the literature as some underlying exchangeability is required. In Bhattacharyya and Bickel^16^, the exchangeability is given through exchangeable weights on regular dependency graphs, while Holmes and Reinert^17^ consider exchangeable network models only. Here we theoretically motivate and exploit a link with the bootstrap method to justify our use of samples of ego-networks to estimate Netdis.

Our empirical results indicate that for several experimental and random model-based datasets, sampling only a small fraction of ego-networks gives comparable performance to the use of Netdis with all possible ego-networks. The observation stands true when dealing with very large graphs and thus makes Netdis applicable to a much wider range of data domains compared to other existing methods. Furthermore, the sampling procedure does not require the networks to be fully known. For instance, in the case of very large networks, such as the WWW, obtaining a sample of ego networks is much more feasible than capturing the entire network. In other cases, obtaining the full network might be too costly, for instance in networks of sexual contacts, and obtaining samples of ego networks might prove to be more straightforward. Together, these improvements make Netdis suitable for challenging datasets that are not amenable to analysis by other methods.

## Datasets

For this study, we used the datasets from Ali *et al*.^8^ for benchmarking, as well as much larger simulated networks. Detailed descriptions of the data sets and reference clustering are provided below. To assess the performance of the network comparisons a reference or ‘ground truth’ clustering is required which groups similar networks into clusters.

### Synthetic networks from random graphs models

For the synthetic networks we consider six random graph models, namely: Erdös-Rényi random graphs^18^, the configuration model^19^,^20^,^21^, 3D geometric random graphs^22^,^23^, geometric random graphs with gene duplication^24^, the Chung-Lu model^25^ and the duplication-divergence growth model^26^.

The first set of synthetic networks we consider consists of 30 simulated networks, where five samples were generated from each of the six random graph models for which the parameters were chosen such as to replicate the number of edges and nodes and whenever the models allow for this also the degree distribution of the DIP core yeast interaction dataset^27^ as closely as possible. The DIP core yeast interaction dataset is a high quality set of protein interactions and the version used in this paper (dated: 2012-02-28) contains 2,160 nodes and 4,300 edges.

In addition to the data set described above we also consider several sets of synthetic networks consisting of larger networks i.e. 10,000, 25,000, 50,000 and 100,000 nodes. We did not consider the Chung-Lu model for these data sets since for large networks the model is expected to produce networks which are very similar to the configuration model. For these data sets the model parameters were chosen such that the resulting networks have an average degree of approximately 20. The degree sequence of the configuration model was chosen to be the degree sequence of one of the duplication diverge networks from the same data set. A more detailed description of the random graph models and data sets is given in the Supplementary material.

For synthetic networks, the correct classification is deemed to be the one that clusters networks from the same model together.

The use of random graphs inevitably introduces some variation in the results obtained by Netdis. In order to verify that the behaviour under sub-sampling does not depend on the data set under consideration we generated 25 independent copies of the first data set and 5 independent copies for each of the data sets containing larger networks. The analysis of these data sets of which the results can be found in the Supplementary material show that our results vary only slightly between independent data sets and that the observed behaviour under sub-sampling is qualitatively the same for all of them.

### Protein-protein interaction (PPI) data

We downloaded species-specific PPI data from the Database of Interacting Proteins (DIP)^28^ and Human Protein Reference Database (HPRD)^29^. Only species having at least 500 physical interactions and >15% coverage were considered. Coverage is here a rough estimate of how many proteins have been probed for interactions given the expected proteome of the organism. We define it as a percentage by taking the number of nodes in the network divided by the estimated number of genes in the genome of the organism at hand. In total, we analyse five species: *Saccharomyces cerevisiae* (yeast), *Drosophila melanogaster* (fly), *Homo sapiens* (human), *Escherichia coli* (ecoli) and *Helicobacter pylori* (hpylori). The networks range in size from 714 nodes to 9673 nodes and in density from 0.0008 to 0.0053. To be able to compare results with Ali *et al*.^8^, we used archived versions of the data rather than the latest available data for these species. Human data came from HPRD (dated: September 2012) while the other four data sets were downloaded from DIP (dated: 2012-02-28). For this PPI dataset the correct classification is assumed to be the species tree from the NCBI taxonomy database^30^.

### Networks from multiple disciplines

Onnela *et al*.^31^ constructed a taxonomy of a large collection of networks obtained from a variety of sources. Their method is based on first probing the community structure within each network and then using summaries of the community structures to identify similar networks. The original dataset used by the authors contains 746 networks. We used all unweighed and undirected networks from this set, resulting in a total of 151 networks. These networks come from across the biological and social domains as well as model simulations. The data is highly variable in size (30 to 11586 nodes) and density (0.0004 to 0.5). For this dataset, the correct classification is deemed to be the one that clusters networks by domain (see Ali *et al*.^8^ for more details).

## Results

The results presented here were obtained using subgraphs of size 4 and the DIP-core yeast interaction network for centering subgraph counts (see Methods). To test the sensitivity of the performance of Netdis under subsampling, the same analysis was also performed using an Erdös-Rényi random graph with 5,000 nodes and 50,000 edges as the reference data set and produced similar results which are given in the Supplementary material. Such a sensitivity analysis was also carried out on the performance of Netdis in Ali *et al*.^8^.

### Nearest neighbour scores

The behaviour of Netdis under sub-sampling is assessed by comparing the nearest neighbour assignments of networks according to Netdis with a ‘ground truth’ or ‘reference’ clustering. A network is said to have a correct nearest neighbours whenever its nearest neighbours are in the same cluster as the network. For example, in the synthetic data sets the nearest neighbour of a geometric random graph should be another geometric random graph and not an Erdös-Rényi random graph.

We quantify the overall performance of Netdis on a given data set by checking the nearest neighbours of networks. We define the nearest neighbour score (1 − *NN*) to be the fraction of correctly assigned nearest neighbours. We refine this score to include a larger number of nearest neigbours. In the ground truth clustering, clusters may be of different sizes and for a *k*-nearest neighbour score to make sense *k* needs to be smaller than the smallest cluster. Therefore, in the latter variant of the score *k* takes different values for different networks so that *k* = *C*_*G*_ − 1, where *C*_*G*_ is the size of the cluster to which *G* belongs. This score, which we call *k*_*C*_ − *NN*, is the fraction of correctly assigned neigbours in the ‘ground truth’ cluster of the network. For *k*_*C*_ − *NN*, a perfect score of 1 is attained only when the largest separation between any two networks within a cluster is smaller than the smallest separation to any of the networks that are not in the same cluster. While the 1 − *NN* score can be thought of as measuring whether networks in the same cluster are located in the same local neighbourhood (or how closely networks in the same cluster are positioned), the *k*_*C*_ − *NN* score measures how well clusters are separated from each other.

### Effect of sampling ego-networks

**Figure 1:**
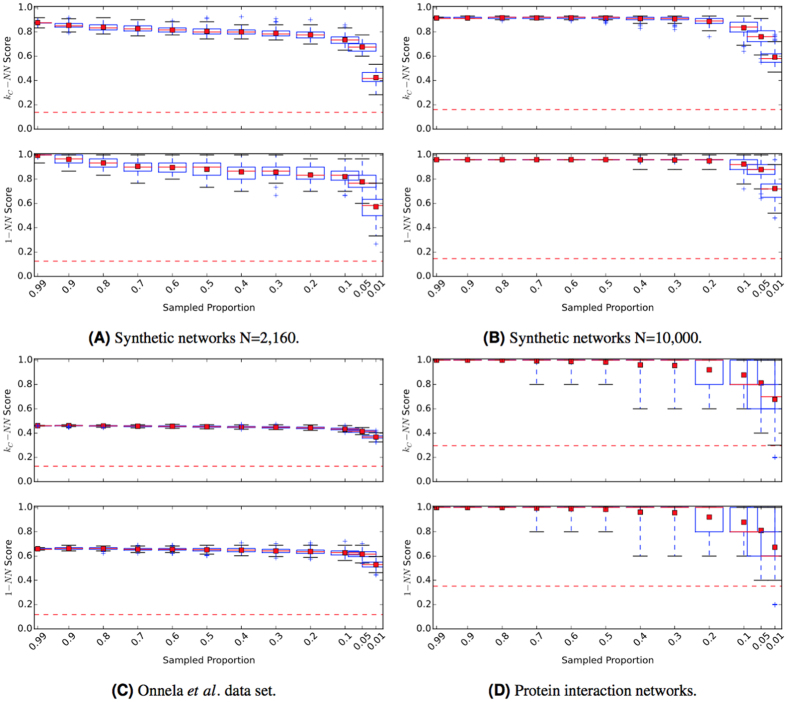
Effect of ego-network sampling on Netdis performance measured by nearest neighbour scores 1 − *NN* and *k*_*C*_ − *NN*: (**A**) Simulated networks from different random graph models with model parameters matching the DIP-core yeast network with 2,160 nodes, (**B**) Simulated networks from different random graph models with 10,000 nodes and average degree ≈20, (**C**) Onnela et al. data containing 151 networks of sizes ranging from 30 to 11586 nodes and (**D**) Protein interaction networks of *Saccharomyces cerevisiae* (yeast), *Drosophila melanogaster* (fly), *Homo sapiens* (human), *Escherichia coli* and *Helicobacter pylori*. The dashed red lines correspond to the average nearest neighbour scores over a sample of 50 random distance matrices. The performance of the Netdis statistics starts to deteriorate strongly only when less than 10% of the ego-networks of each network are sampled. Even when only 1% of the ego-networks are sampled the statistics performs better than the random baseline.

Netdis performs well in classifying networks by model type correctly even with networks of highly different sizes and densities. However, this ability comes at a computational cost; subgraphs must be counted in each ego-network of the complete network which can be prohibitive for very large networks (e.g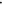 >100,000 nodes). We thus investigate here whether the Netdis statistic can be based on only a sample of the ego-networks by calculating the sum of centered subgraph counts (see the Methods section) over a randomly chosen subset of ego-networks in each network. The effect of the sampling ratio on the ability of the method to correctly classify networks is shown in Fig. 1. The vertical axis represents the level of agreement between the ideal clustering of networks and the one generated by Netdis, measured by the nearest neighbour score. The horizontal axis represents the percentage of ego-networks sampled from each network in the dataset (250 repetitions were carried out to generate the box-plots).

As expected, in general, sub-sampling of ego-networks leads to a decrease in performance. However, the method performs surprisingly well on average even when sampling only around 10% of the ego-networks in each network. The performance falls off drastically only below 5% sampling. While the results in Fig. 1A,B are based on simulated networks, similar results are observed for the Onnela *et at*.^31^ data (Fig. 1C) and empirical protein interaction networks (Fig. 1D). Again, the average performance of the method degrades substantially only below 5% sampling despite the fact that the networks in the data vary significantly in size, with some being very small (<150 nodes).

The random baseline in the figures is obtained by taking the average nearest neighbour scores over a sample of 50 random distance matrices.

### Choice of sample size

**Figure 2:**
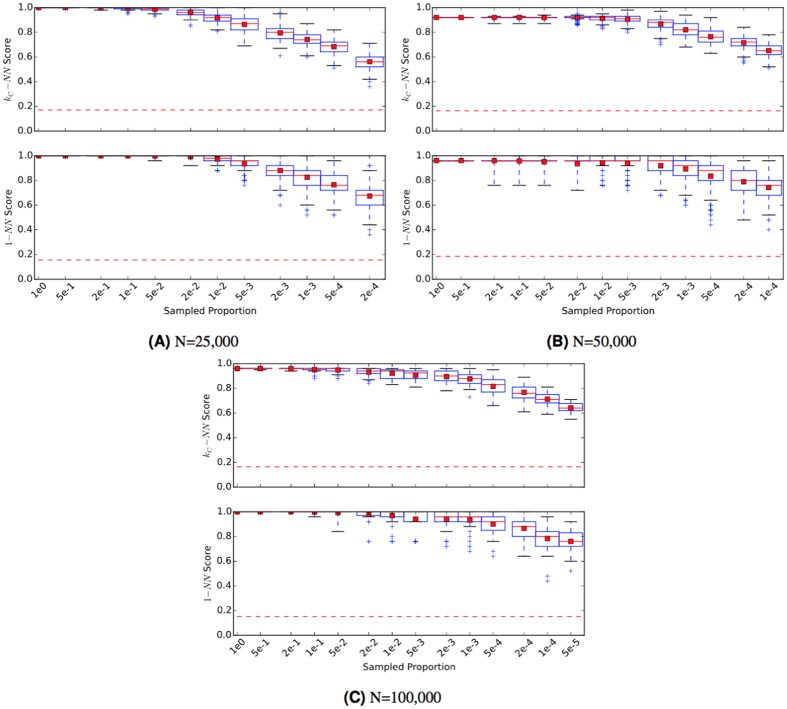
Netdis performance under sub-sampling measured by measured by nearest neighbour scores 1 − *NN* and *k*_*C*_ − *NN* for large simulated network data sets with average degree ≈20 each containing 5 realizations of 5 different random graph models: (**A**) Networks with 25,000 nodes, (**B**) Networks with 50,000 nodes and (**C**) Networks with 100,000 nodes. The dashed red lines correspond to the average nearest neighbour scores over a sample of 50 random distance matrices. Note that the x-axes are scaled logarithmically. While the performance of Netdis slightly deteriorates with smaller sample size, the deterioration is sublinear in the the number of nodes. The *k*_*C*_ − *NN* score is only slightly smaller than the 1 − *NN* score. For networks with 100,000 nodes even sampling only 10 ego-networks contains a strong signal.

An important question is whether an acceptable level of performance of Netdis is related to the proportion of all ego-networks sampled or whether an absolute minimum number of ego-networks is required. For extremely large networks, an indication of the latter case would be a massive computational gain as only a limited number of ego-networks would need to be sampled. We tested this conjecture by carrying out the sub-sampling analysis on larger versions of the simulated network dataset, with networks of 25,000, 50,000 and 100,000 nodes respectively. Our results indicate that for these data sets sampling even a small number of ego-networks often suffices for obtaining close to optimal performance when assessed by *k*_*C*_ − *NN* as well as by 1 − *NN*. This is evident from Fig. 2 where a much smaller sample (≈10 ego-networks) suffices for an almost optimal performance compared to the datasets with networks containing ≈2,000 and 10,000 nodes (Fig. 1). These findings suggest that one can compare very large networks using Netdis even if only a small sample of ego-networks is available.

## Discussion

Analysis and comparison of very large and dense networks is a computational challenge for most existing methods. In this paper, we have presented empirical and theoretical results which justify the use of sampling within Netdis, a distance-based network comparison framework. Netdis originally uses counts of small subgraphs in all ego-networks in a network to define a dissimilarity score. While this scheme was shown to work well for a wide variety of empirical and simulated networks, the linear dependence of the computational cost on the number of nodes in a network remains an issue. Additionally, Netdis assumes that the complete adjacency matrix for the network is available with no missing nodes. An attractive option to circumvent this issue is to base the dissimilarity score only on a small number samples of ego-networks from each network.

Here we have shown that such a sample-based approach does indeed show promising results. In particular, for a variety of network datasets, a surprisingly small sample of ego-networks from each network is sufficient in general to recover the correct classification. Moreover, we observed that the minimum required sample size for acceptable performance scales sub-linearly with network size. Hence, the proposed sampling scheme can improve execution times by orders of magnitude. For instance, analysing the PPI dataset using Netdis without sampling takes 1 hour on a single-processor desktop computer, while the same dataset can be analysed in slightly less than 10 minutes with an ego-network sampling rate of 10% for each network. Another advantage of the sampling approach is that the complete network need not be known for the method to work, potentially allowing networks upwards a million nodes to be compared. As a caveat, for the Onnela *et al*. network data set in this study the “ground truth” was established by a heuristic. The heuristic assumes that data from similar experiments should be closer to each other than data from different experiments. This assumption may be violated for some examples; some college Facebook networks for example may display a very different topology than other college Facebook networks, due to some characteristics of the college which we are not able to access.

The subsampling procedure might fail in certain special cases where one or more networks under consideration contain small regions that differs significantly from the rest of the network simply because such regions might not be represented in the sample. Such an example in which subsampling fails that considers sparse Erdös-Rényi random graphs (N = 10,000, E = 15,000) to which a large complete graph (N = 30) is added can be found in the Supplementary material.

Sub-sampling approaches for networks have to take the dependence structure between nodes (or edges) into account. In this paper we have adapted a bootstrap procedure from Holmes and Reinert^17^ which was developed for random regular graphs. The procedure is based on ego-networks and is therefore ideally suited to sample from ego-networks based network comparison measures. Currently the only such available network comparison statistic is Netdis. It is plausible that a similar sub-sampling scheme could work well for other statistics which are based on ego-networks.

The empirical observations in this paper are bolstered by our theoretical results that justify the use of samples provided they contain ego-networks of comparable sizes. The enhancements and accompanying results presented here should make Netdis applicable to domains with typically very large networks (e.g. social or telecommunication networks with upwards of a million nodes) while still maintaining the superior performance of the method’s many-to-many comparison approach.

## Methods

### Brief overview of Netdis

The Netdis statistic measures the dissimilarity between a given pair of networks. The statistic is based on the counts of small *k*-node subgraphs in the two-step ego-networks of the two networks. The two-step ego-network of a protein/node *p* is the (sub) network consisting of all nodes within two edges of *p*, where we also include all the edges between these nodes. To compare two networks, *G* and *H*, we first calculate the sum of centered counts of subgraphs over all ego-networks in each network, denoted by *S*_*w*_(*G*) and *S*_*w*_(*G*). The counts are centered by first subtracting the expected number of counts found in the ego-networks of a reference model/dataset having similar density (see Ali *et al*.^8^ for more details). In this article, we use the DIP-core yeast interaction network for centering. Then the 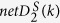 statistic is defined as


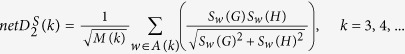


where *A*(*k*) is the set of all small graphs on *k* nodes (so that *A*(3) has 2 elements and *A*(4) has 6 elements) and


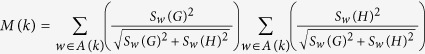


is a normalizing constant so that 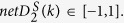 by the Cauchy-Schwarz inequality. The corresponding Netdis statistic is:


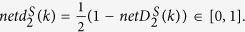


The crucial idea introduced by Netdis is the use of counts from all ego-networks of a network, thus enabling a many-to-many comparison between two networks. For computational reasons Netdis usually takes *k* = 4.

### Theoretical underpinning of the sampling scheme

The use of the sub-sampling scheme developed in this paper relies on the assumption that the dissimilarity scores calculated from samples converge to the true Netdis score. We justify the sub-sampling theoretically by generalizing a result of Holmes and Reinert^17^ concerning bootstrapping in dependency graphs. This result was shown for random regular graphs only, so that each node in the dependency graph has the same degree. In a dependency graph nodes have random variables attached to them and two nodes are connected by an edge if the associated random variables are dependent. The example given in Holmes and Reinert^17^ is based on a network where every node has the same degree, and there are independent random weights on the edges of this network. A node is attributed the sum of the weights of the edges which have this node as an endpoint. Then the node sums for two nodes *i* and *j* are dependent if and only if *i* and *j* are connected by an edge. Hence the dependency graph coincides with the network.

For the networks which we consider we do not assume that the dependency graph coincides with the network. Instead we consider the dependency graph on the same node set as the network, but connecting two nodes if they are at most two network edges away from each other, so that they are contained in each other’s two-step ego-network. This assumption is motivated by the view of networks being composed of small functional units.

For example, perhaps we are interested in the number of triangles in a Erdös-Rényi random graph. From the Erdös-Rényi random graph we generate a new graph which has triples (*i*, *j*, *k*) as nodes; the random variable on the triples is the indicator that the triple forms a triangle. Then two triples are connected by an edge if they share at least one element. This dependency graph would be a regular graph; each triple has 
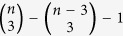
 neighbours (which is of the order *n*^2^).

More related to Netdis, we may be interested in the random vector containing the number of small graphs on three nodes in one-step ego-networks in a Erdös-Rényi random graph. From the Erdös-Rényi random graph we generate a new graph which has the same nodes as the original random graph; the random variable on the nodes is the random vector containing the number of small graphs on three nodes in its one-step ego-network. Then two nodes are connected by an edge if their one-step ego-networks overlap. For each node *v*, the set of all other nodes for which their one-step ego-networks overlap with the node *v* is just the two-size ego-network of *v*. This dependency graph would in general not be a regular graph.

More generally, we start with a dependency graph on a population of *N* nodes which is constructed as follows. Each node has a random element *X*_*i*_ associated with it. Two nodes *i* and *j* are linked by an edge (denoted by *i* ~ *j*) if and only if their corresponding random elements *X*_*i*_ and *X*_*j*_ are dependent. The neighbourhood of dependence for the random variable *X*_*i*_ is given by *S*_*i*_ = {*j* : *i* ~ *j*, *j* ≠ *i*}, and we let *γ*_*i*_ = |*S*_*i*_|, *i* = 1, …, *N*, denote their sizes. We then think of the dependency graph generated from the network data on *n* nodes as an induced subgraph sample of size *n* from this larger dependency graph on *N* nodes. Formally, let *n* ≤ *N*, sample *X*_1_, *X*_2_, …, *X*_*n*_, obtaining 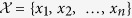. Given this induced subgraph sample, the edges in the sample are no longer random, as they have now been observed. Randomness enters through the bootstrap procedure: we sample 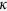 seed nodes from the dependency graph with replacement, and every seed node is equally likely to be in the sample. For each seed node we also include all nodes from its dependency neighbourhood in the sample. Thus, our sampling procedure chooses indices *k*_1_, …, *k*_*n*_ according to the multinomial distribution 
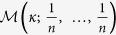
. We throw *κ* balls into *n* boxes independently, with each box being equally likely. If index 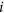 is chosen, then we sample the whole dependency neighbourhood of *X*_*i*_. The bootstrap results are conveniently summarised by an empirical measure, which has point mass at every node in the sample. Each such point mass is weighted proportionally to the number of times that the node is in the sample, standardised such that the total mass of the empirical measure is 1. The above procedure gives rise to the empirical measure


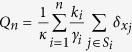


which we would like to compare to the true empirical measure 
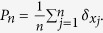
 Here 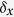 denotes point mass at *x* i.e. *δ*_*x*_(*A*) =1 if *x* ∈ *A* and *δ*_*x*_(*A*) =0 if *x* ∉ *A*. We let


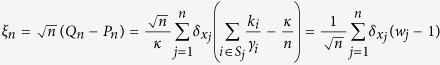


denote the empirical measure for the comparison, where we use as weights 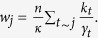 For example, for an integer *l*, 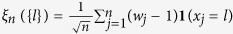, where **1**(*x*_*j*_ = *l*) = 1 if *x*_*j*_ = 1, and 0 otherwise. A bootstrap procedure is said to work if it behaves approximately like a Gaussian random element centred around the true empirical measure. Hence the bootstrap works if the distance between 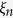 and a centred Gaussian random measure *G*_*mult,deep,samp*_ goes to 0 as *n* → ∞, where *G*_*mult,deep,samp*_ has covariance matrix.


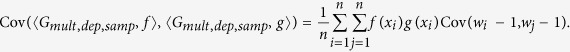


If the neighbourhoods of dependence are small, then the bootstrap should work because there is only weak dependence between the random weights. The distance between the random measure *ξ* and the limiting Gaussian random measure is defined as follows. For 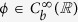, the set of infinitely often differentiable real-valued functions with bounded derivatives of all order, let 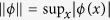 and 
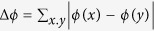
. Here, for a function *f*, *f*_(*j*)_ is the partial derivative of *f* in direction *x*_*j*_, and similarly *f*_(*i*,*j*)_, *f*_(*i*,*j,k)*_ denote higher partial derivatives. The result is phrased in terms of cylinder-type functions 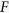 which take measures as input and are of the form


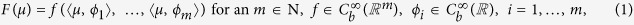


where 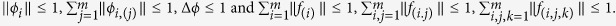


It is shown in ref. 32 that if the random measures converge for test functions of these types, then the measures themselves converge (in the vague topology).

**Proposition 1.** *In the above bootstrap procedure, for all H of the form* (*1*),


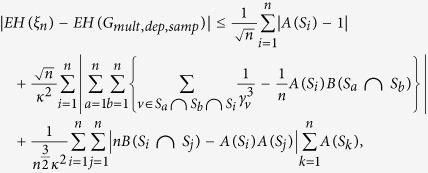


*where* 
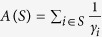
 *and* 
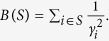


Proposition 1 quantifies the effect of varying neighbourhood sizes. Here we think of *κ* as having similar magnitude as *n*. The bound is not claimed to be sharp but instead indicative for when the procedure works - the individual neighbourhoods should not vary too much in size, and the overlap between neighbourhoods should not be too large on average. The proof of Proposition 1 can be found in the Supplementary information.

For illustration of the bound, suppose that there are *M* neighbourhoods which are “unbalanced” in the sense that *A*(*S*) ≠ 1. Suppose that *γ* is the typical value for the neighbourhoods and let 
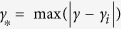
. Then the bound is roughly of the order 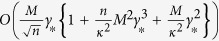.

The typically largest contribution to the bound is the term 
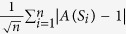
, which originates from *Ew^i^* = *A*(*S_i_*) which may not equal 1. Using an approximation with the same Gaussian centered random measure but approximating instead of *ξ*_*n*_ the empirical measure 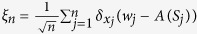 would give a smaller overall bound on the approximation error; only the second and the third summands would appear. Whilst the proposition provides a set of conditions which guarantee the subsampling to work asymptotically, deviations from these do not necessarily imply that the procedure will fail.

However, in the Supplementary information, we provide an example in which the conditions of the proposition are strongly violated and the subsambling procedure performs poorly. This example concerns an Erdös-Rényi random graph (N = 10,000, E = 15,000) which contains an additional disconnected complete graph on 30 nodes. The ego networks corresponding to the disconnected complete graph are of size 30 compared to an average of 10 for the 2-step Erdös-Rényi random graph. Moreover, the 2-step ego-networks in the disconnected component are all identical and intersect completely.

When applying the graph bootstrap procedure to Netdis we carry out a leap of faith by assuming that two-step ego-networks correspond to edges in the dependency graph. Indeed it could be interesting to modify the bootstrap procedure to include three-step or four-step ego-networks. In the data sets which we considered, three-step ego-networks often cover a large proportion of the entire network, and hence there is no computational gain from the bootstrapping procedure.

### Data availability

An implementation of Netdis is freely available at http://www.stats.ox.acuk/research/proteins/resources.

## Additional Information

**How to cite this article**: Ali, W. *et al*. Comparison of large networks with sub-sampling strategies. *Sci. Rep.* **6**, 28955; doi: 10.1038/srep28955 (2016).

## Supplementary Material

Supplementary Information
