# Supplementary material for: Comparison of large networks with sub-sampling strategies
Source: Sci Rep. 2016 Jul 6;6:28955. doi: 10.1038/srep28955 (PMC4933923; doi:10.1038/srep28955)
Supplement: Supplementary Information [file srep28955-s1.pdf]

Supplementary Information: Comparison of large networks with sub-sampling strategies.

Waqar Ali[1], Anatol E. Wegner[1,*], Robert E. Gaunt[1], Charlotte M. Deane[1], and Gesine Reinert[1]

[1]Department of Statistics, University of Oxford, 1 South Parks Road, Oxford OX1 3TG, UK.

*wegner@stats.ox.ac.uk

Synthetic network data sets

Synthetic Networks Data Set 1

These 25 independent data sets consist each of 30 simulated networks corresponding to five realizations from each of the following random graph models, namely: namely: Erdős-Rényi random graphs with fixed number of edges [1], the configuration model [2–4], 3D geometric random graphs [5, 6], geometric random graphs with gene duplication [7], the Chung-Lu model [8] and the duplication-divergence growth model [9]. Each network was grown to the size of the DIP-core yeast interaction data set and the parameter of the models were chosen such as to replicate the number of edges of the DIP-core yeast network as closely as possible. The DIP-core network has 2160 nodes and 4300 edges. Whenever the models require the degree distribution to be specified, the degree distribution of the DIP-core yeast interaction data set network was used.

Synthetic Networks Data Set 2

These data sets consist each of 25 simulated networks corresponding to five realizations from each of the following random graph models, namely: Erdős-Rényi random graphs with fixed number of edges, the configuration model, geometric random graphs, geometric random graphs with gene duplication and the duplication-divergence growth model. Networks were grown to the sizes of 10,000, 25,000, 50,000 and 100,000 nodes (5 independent data sets for each case except the data set of networks of 100,000 nodes because of computational limitations) and parameters of the models were chosen such as to obtain networks with an approximate average degree of 20. In the case of the configuration model the degree distribution of one of the duplication divergence networks from the data set was used. The number of edges and nodes fluctuate slightly because not all models allow the number of edges to be fixed and some contain significant numbers of isolated nodes which are discarded during preprocessing.

Results for independent data sets

Here we present results for the additional data sets of synthetic networks.

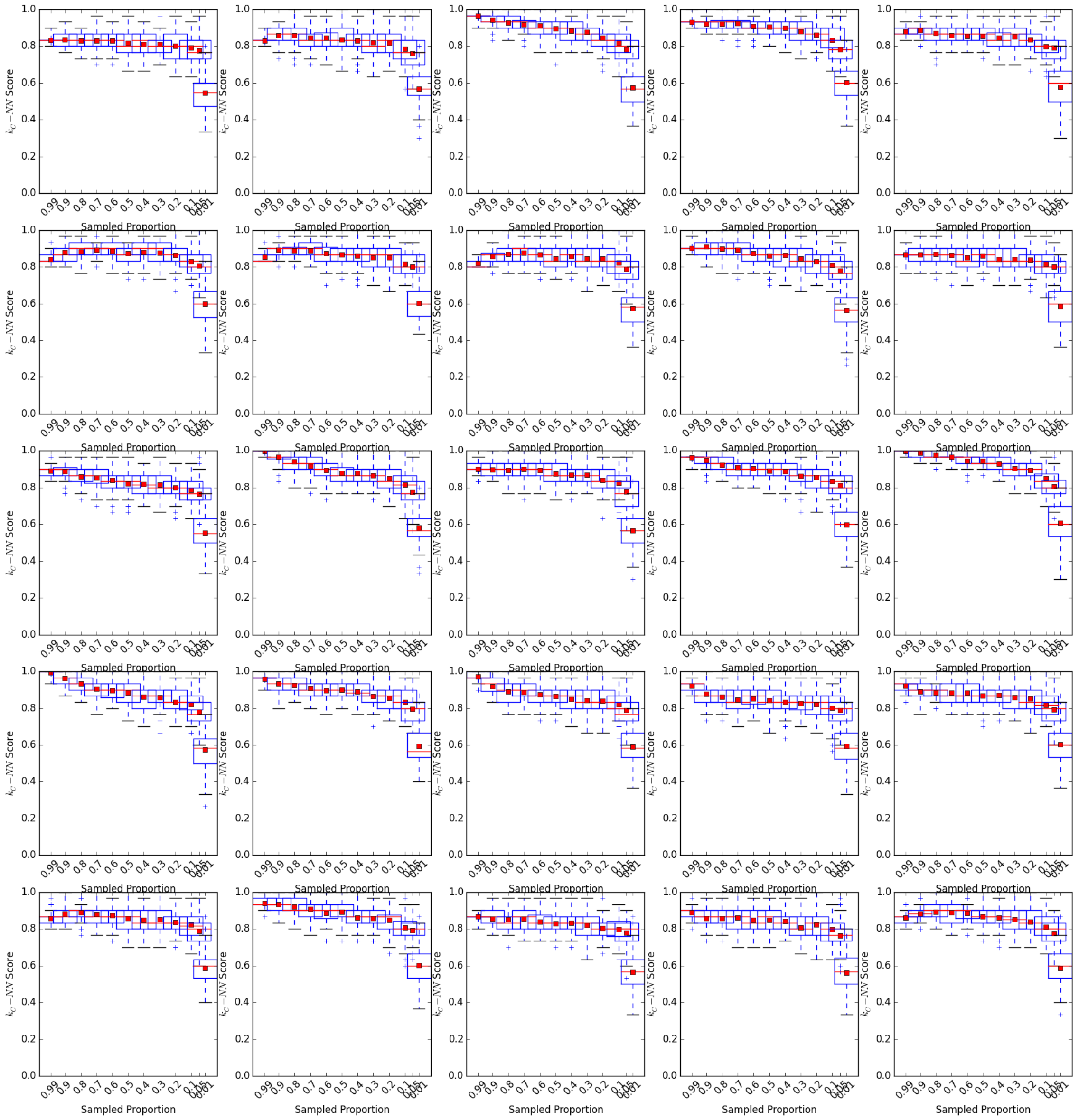

Figure 1: Netdis performance as measured by the $k_C - NN$ score under sub-sampling for 25 independent copies of synthetic networks data set 1 with 2160 nodes.

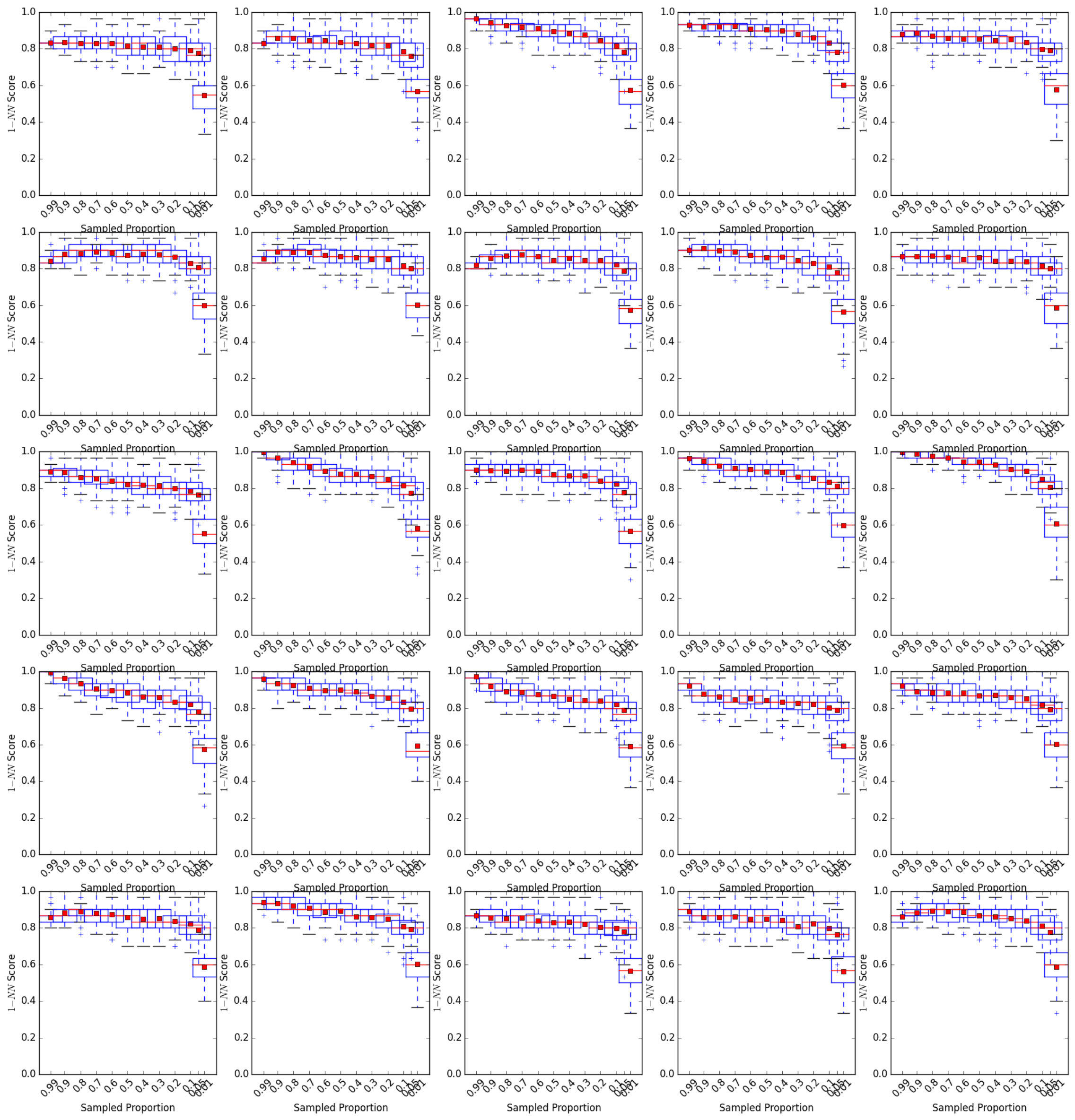

Figure 2: Netdis performance as measured by the $1-NN$ score under sub-sampling for 25 independent copies of synthetic networks data set 1 with 2160 nodes.

The variations of the results between the data sets in Fig.1 and 2 come mostly from the mixing between the configuration model and the Chung-Lu models. This mixing is not surprising given that these two models converge for large networks. Another source of variation is the duplication divergence model which in certain realizations produces non typical networks. Again, this is not surprising since the duplication divergence model is known to be quite unstable [10]. For instance, the same parameter values can produce networks that differ significantly with respect to the number of edges.

The results given in Figures 3, 4 and 5 show that the qualitative behaviour of Netdis under sub-sampling is the same for all independent data sets we considered.

Results with Erdös-Rényi background

Figures 6 and 7 show the results obtained using an Erdös-Rényi random graph with 5,000 nodes and 50,000 edges as the gold standard in Netdis.

Although we observe slight variations in the starting points of the plots when compared to the use of the DIP-core yeast interaction network as a gold standard, the results show that the behaviour of Netdis under sub-sampling is not significantly affected by the choice of gold standard network. The most notable of these variations occurs in the case of the protein interaction dataset where the initial NN score (i.e. the score without sampling) is 0.6 compared to 1.0 when the DIP-core network was used as a gold standard. Despite this variation we still observe that the performance of Netdis degrades significantly only below 10% sampling probability when compared to the performance without sub-sampling.

In the case of data sets containing large networks using an Erdös-Rényi random graph as a gold standard decreases the overall $1 - NN$ and $k_C - NN$ scores for small sample sizes compared to the DIP-core gold standard but one still gets a strong signal even when as few as 10 ego-networks are sampled.

An example where sub-sampling fails

Here we consider a data set that consists of 5 Erdös-Rényi random graphs on 10,000 nodes with 15,000 edges and 5 Erdös-Rényi random graphs on 10,000 nodes with 15,000 edges to which a disconnected complete graph of size 30 is added. For this data set we the nearest neighbour scores start to deteriorate much quicker when compared to other data sets and the signal is almost completely absent for sampling probabilities as high as 0.5. Plots for the $1 - NN$ and $k_C - NN$ scores are given in Figure 8.

2-step Ego-network Size Distributions

In order to check that the observed performance of Netdis under sub-sampling is not due to 2-step ego-networks typically covering most of the network we computed the size distributions of 2-step ego networks. Figure 9 shows the size distribution for the 50,000 node synthetic networks data set. We observe that even for networks with scale free degree distributions typical 2-step ego networks do not cover most of the network. Similar results hold for the protein interaction networks where typical two step ego networks span less than 5% of the network.

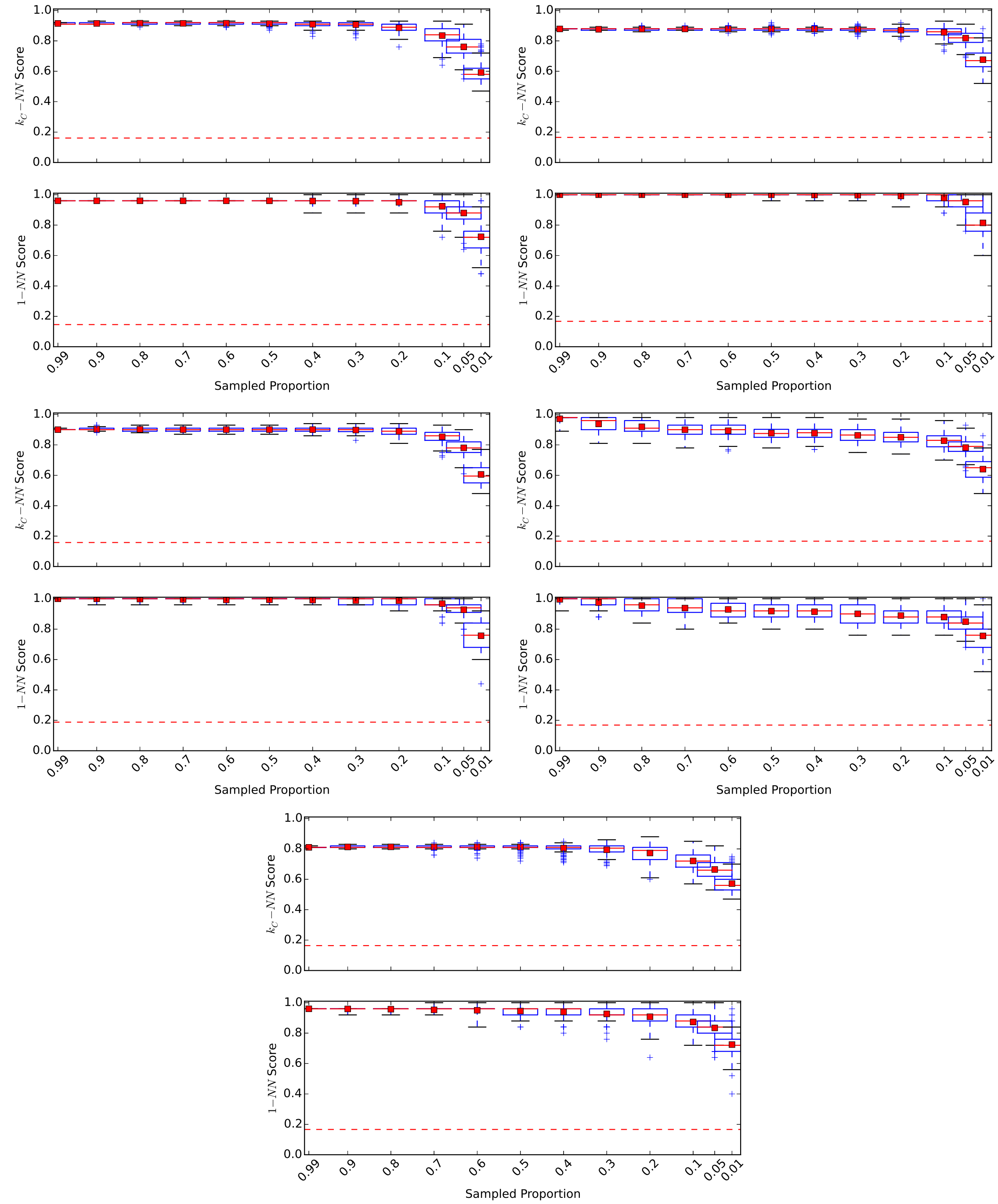

Figure 3: Netdis performance under sub-sampling measured by the fraction of correctly assigned nearest neighbours ($1-NN$ and k_C-NN scores) for 5 independent data sets consisting of synthetic networks of size 10,000. The dashed red lines correspond to the average nearest neighbour scores over a sample of 50 random distance matrices.

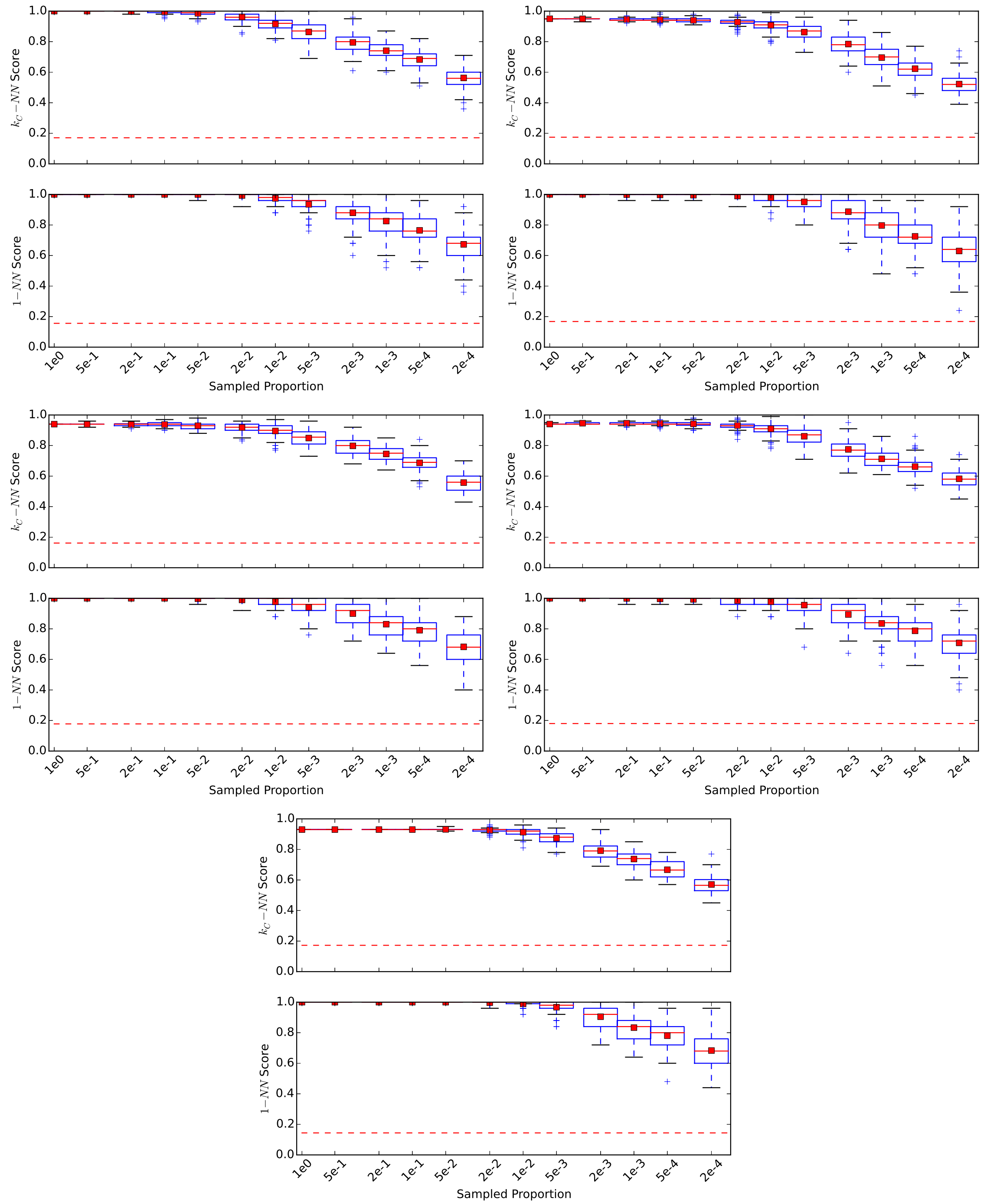

Figure 4: Netdis performance under sub-sampling measured by the nearest neighbour scores $1-NN$ and k_C-NN for 5 independent data sets consisting of synthetic networks of size 25,000.The dashed red lines correspond to the average nearest neighbour scores over a sample of 50 random distance matrices.

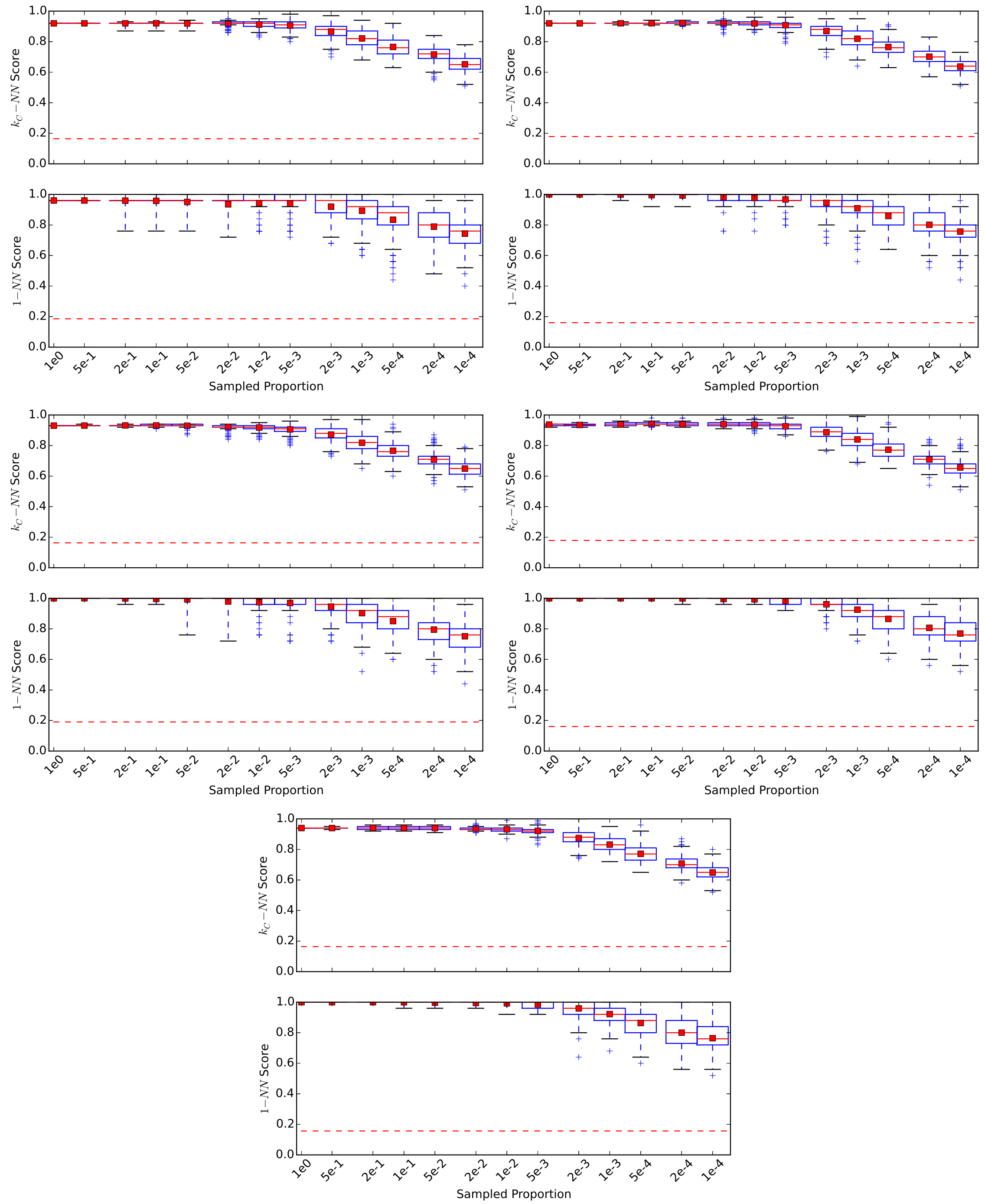

Figure 5: Netdis performance under sub-sampling measured by nearest the neighbour scores $1-NN$ and k_C-NN for 5 independent data sets consisting of synthetic networks of size 50,000. The dashed red lines correspond to the average nearest neighbour scores over a sample of 50 random distance matrices.

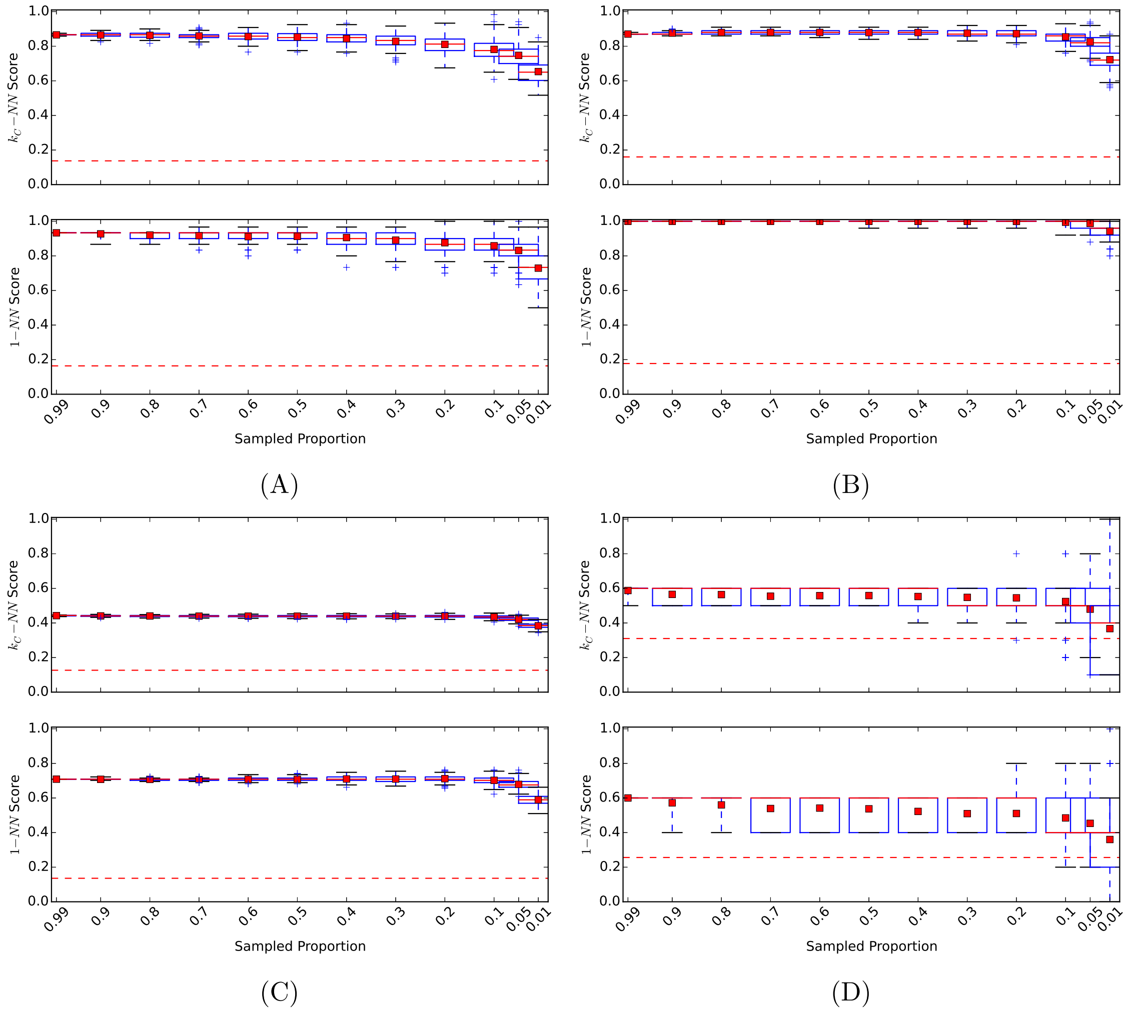

Figure 6: Effect of ego-network sampling on Netdis performance measured by the nearest neighbour scores $1-NN$ and k_C-NN with an Erdös-Rényi random graph with 5,000 nodes and 50,000 edges as a gold standard: (A) Simulated networks from different random graph models with model parameters matching the DIP-core yeast network with 2,160 nodes, (B) Simulated networks from different random graph models with 10,000 nodes and average degree ≈ 20, (C) Onnela et al. data containing 151 networks of sizes ranging from 30 to 11586 nodes and (D) Protein interaction networks of *Saccharomyces cerevisiae* (yeast), *Drosophila melanogaster* (fly), *Homo sapiens* (human), *Escherichia coli* and *Helicobacter pylori*. The dashed red lines correspond to the average nearest neighbour scores over a sample of 50 random distance matrices.

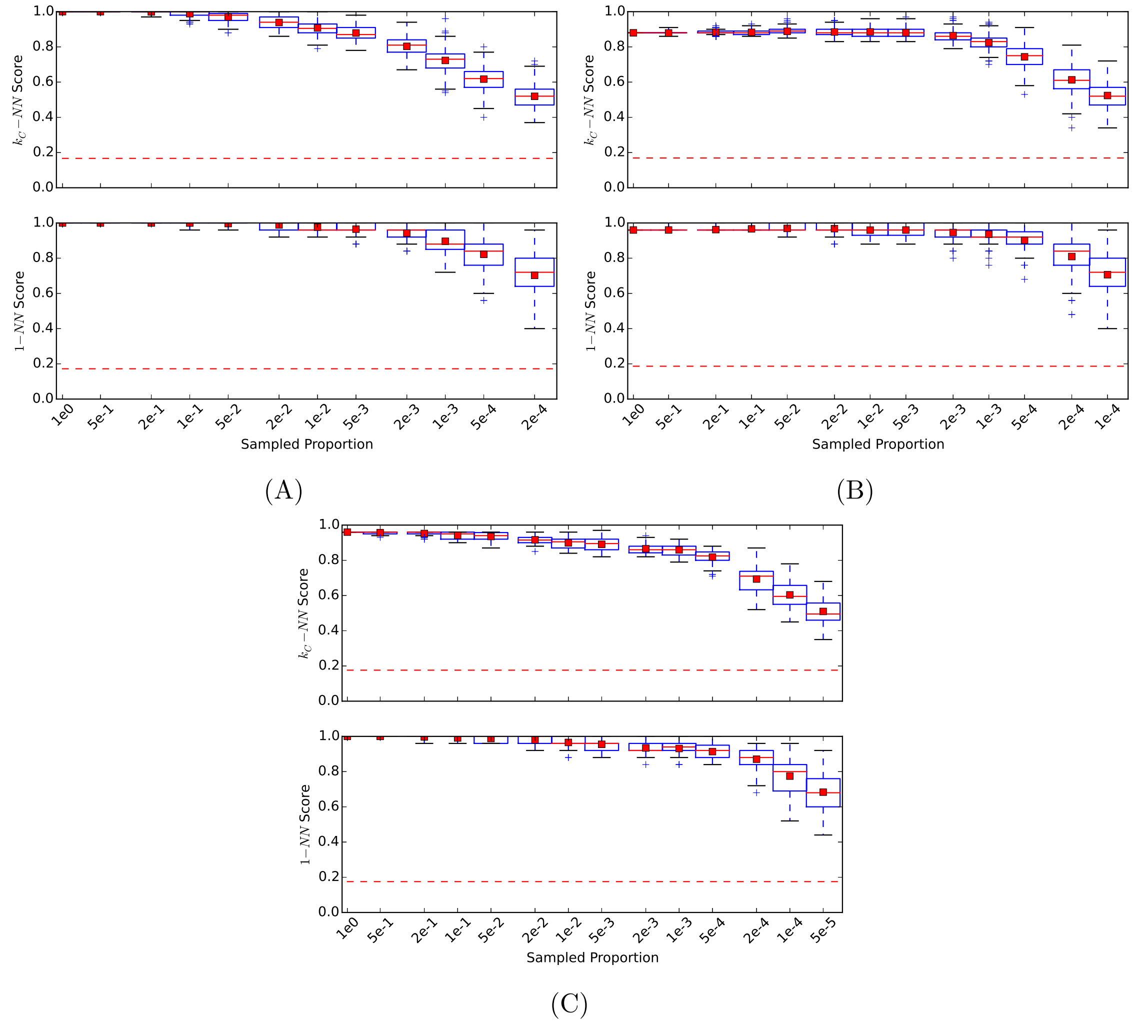

Figure 7: Netdis performance under sub-sampling measured by the nearest neighbour scores $1-NN$ and k_C-NN for large simulated network data sets with average degree ≈ 20 when an Erdös-Rényi random graph with 5,000 nodes and 50,000 edges is used as a gold standard: (A) Networks with 25,000 nodes, (B) Networks with 50,000 nodes and (C) Networks with 100,000 nodes.The dashed red lines correspond to the average nearest neighbour scores over a sample of 50 random distance matrices. Note that the x-axes are scaled logarithmically.

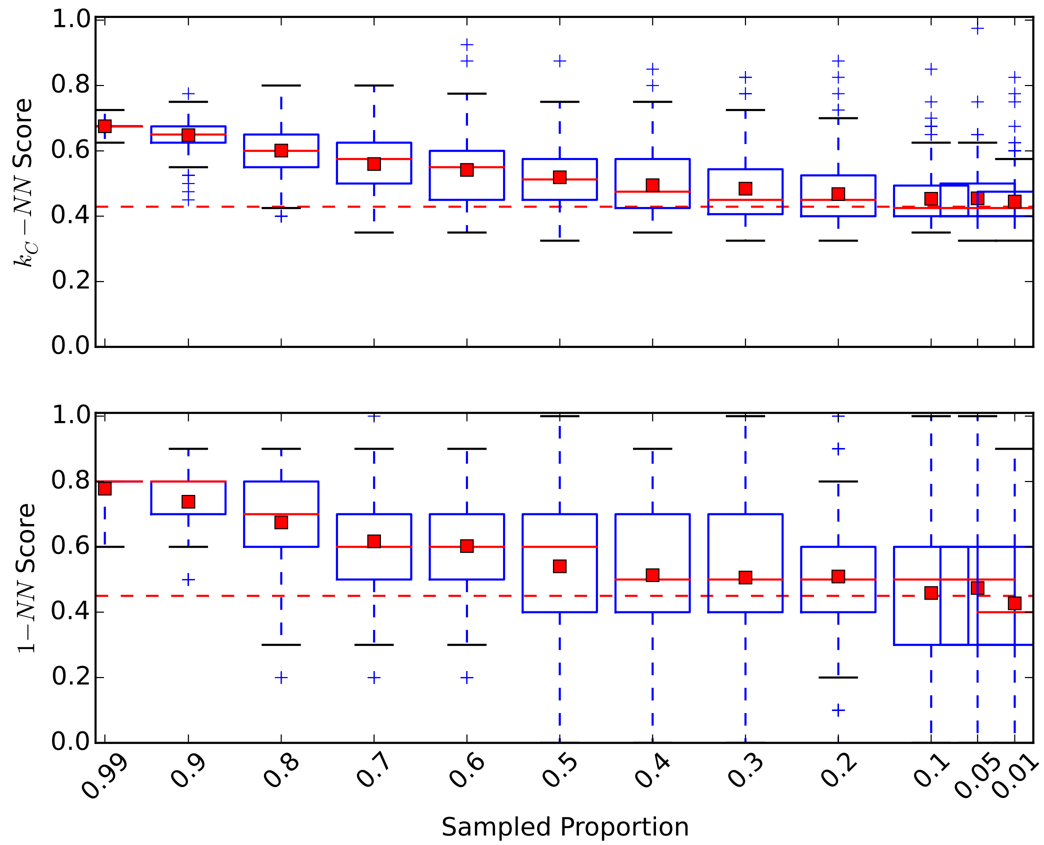

Figure 8: Netdis performance under sub-sampling measured by the nearest neighbour scores $1-NN$ and k_C-NN for a data set consisting of 5 Erdös-Rényi random graphs on 10,000 nodes with 15,000 edges and 5 Erdös-Rényi random graphs on 10,000 nodes with 15,000 edges to which a disconnected complete graph of size 30 is added. The dashed red lines correspond to the average nearest neighbour scores over a sample of 50 random distance matrices. Here, the assumptions of Proposition 1 are strongly violated and the performance of Netids under sub-sampling is poor. This is because the two-step ego networks in the disconnected complete graph are of size 30 are all identical and interest completely, and are of a different size to those typically found in the Erdös-Rényi random graph.

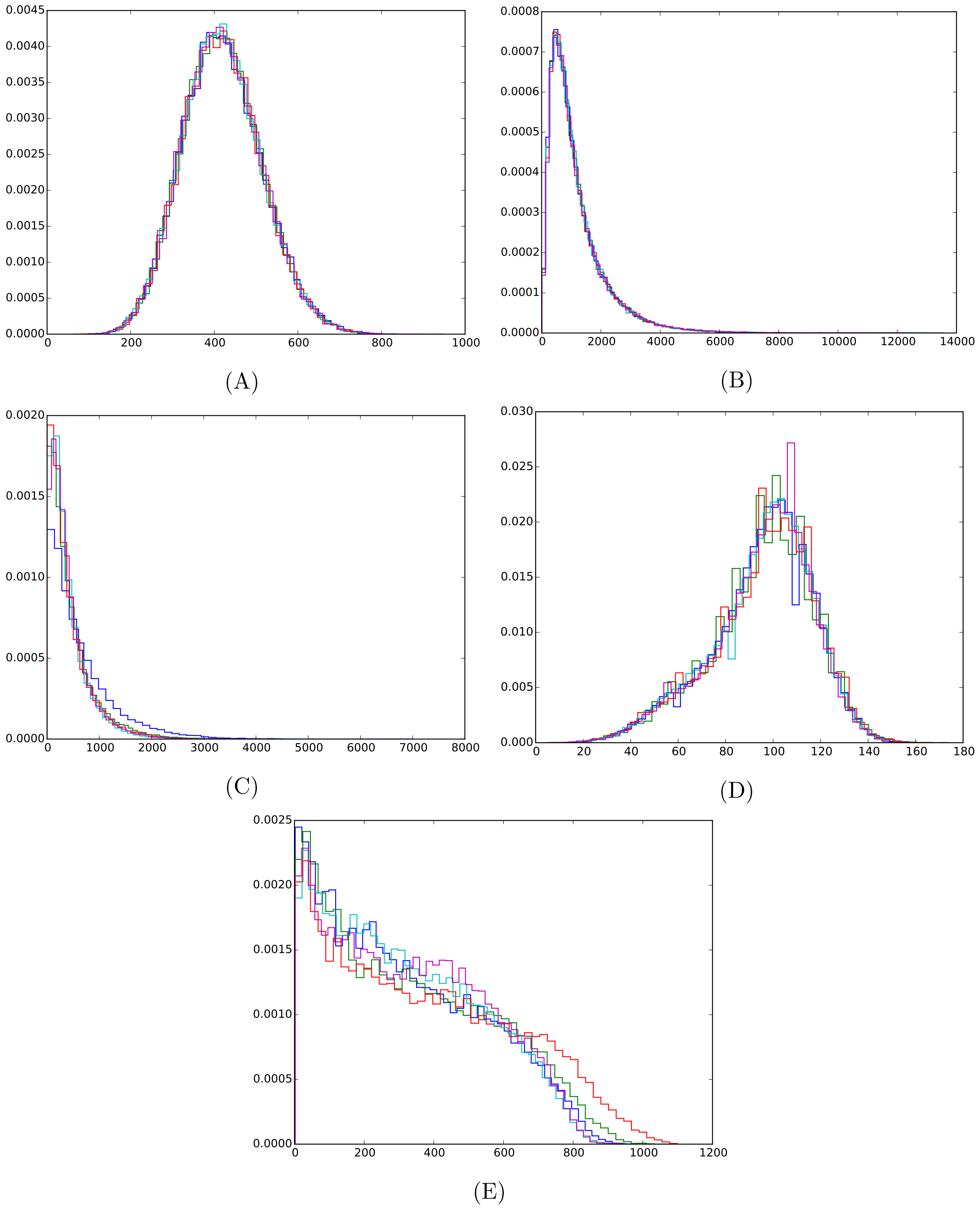

Figure 9: The size distribution of 2-step ego-networks for different random graph models in the first 50,000 node synthetic network data set: (A) Erdös-Rényi model, (B) configuration model,(C) duplication divergence model, (D) 3D geometric random graphs and (E) geometric random graphs with gene duplication. Each histogram shows 5 different realizations for each model.

Proof of Proposition 1.

Here we shall provide a proof of Theorem 0.1. Recall the setting:
The neighbourhood of dependence for the random variable X_i is given by $S_i = \{j : i \sim j, j \neq i\}$, and we let $\gamma_i = |S_i|$, $i = 1, \ldots, N$, denote their sizes. Our sampling procedure chooses indices $k_1, \ldots, k_n$ according to the multinomial distribution $\mathcal{M}\left(\kappa; \frac{1}{n}, \ldots, \frac{1}{n}\right)$. We throw k balls into n boxes independently, with each box being equally likely. If index i is chosen, then we sample the whole dependency neighbourhood of X_i. We study the empirical measure

$$\xi_n = \frac{1}{\sqrt{n}} \sum_{j=1}^{n} \delta_{x_j}(w_j - 1),$$

where $w_j = \frac{n}{\kappa} \sum_{t \sim j} \frac{k_t}{\gamma_t}$. We compare this measure to a centred Gaussian random measure $G_{mult,dep,samp}$ with the corresponding covariance matrix

$$\mathrm{Cov}(\langle G_{mult,dep,samp}, f\rangle, \langle G_{mult,dep,samp}, g\rangle) = \frac{1}{n} \sum_{i=1}^{n} \sum_{j=1}^{n} f(x_i) g(x_i) \mathrm{Cov}(w_i - 1, w_j - 1).$$

In Lemma we shall show that with the notation

$$A(S) = \sum_{i \in S} \frac{1}{\gamma_i} \quad \text{and} \quad B(S) = \sum_{i \in S} \frac{1}{\gamma_i^2}$$

we have

$$\mathrm{Cov}(\langle G_{mult,dep,samp}, f\rangle, \langle G_{mult,dep,samp}, g\rangle) = \frac{1}{n\kappa} \sum_{i=1}^{n} \sum_{j=1}^{n} \Big(nB(S_i \cap S_j) - A(S_i)A(S_j)\Big) f(x_i) g(x_j).$$

We quantify the distance between the random measure ξ_n and the limiting Gaussian random measure in terms of cylinder-type functions F which take measures as input and are of the form

$$F(\mu) = f(\langle \mu, \phi_1 \rangle, \ldots, \langle \mu, \phi_m \rangle) \text{ for an } m \in \mathbb{N},\ f \in C_b^\infty(I\!R^m),\ \phi_i \in C_b^\infty(I\!R),\ i = 1, \ldots, m. \tag{1}$$

For $\phi \in C_b^\infty(I\!R)$, the set of infinitely often differentiable real-valued functions with bounded derivatives of all order, let $||\phi|| = \sup_x |\phi(x)|$ and $\Delta\phi = \sum_{x.y} |\phi(x) - \phi(y)|$. Define the sets

$$\mathcal{C} = \{\phi \in C_b^\infty(I\!R) \text{ with } ||\phi|| \leq 1,\ \sum_{j=1}^{m} ||\phi_{(j)}|| \leq 1,\ \Delta\phi \leq 1\} \tag{2}$$

and

$$\begin{aligned} \mathcal{F} \ := \ \Big\{ & F \in C_b(M^f(I\!R)) : F \text{ has the form (1) for an } m \in \mathbb{N},\ f \in C_b^\infty(I\!R^m) \qquad (3) \\ & \text{with } \sum_{i=1}^{m} ||f_{(i)}|| \leq 1,\ \sum_{i,j=1}^{m} ||f_{(i.j)}|| \leq 1,\ \sum_{i,j,k=1}^{m} ||f_{(i,j,k)}|| \leq 1, \\ & \text{and } \phi_i \in \mathcal{C}, i = 1, \ldots, m \Big\} \end{aligned}$$

Here $f_{(j)}$ is the partial derivative of f in direction x_j, and similarly $f_{(i,j)}, f_{(i,j,k)}$ denote higher partial derivatives. It is shown in [11] that this class of functions is convergence-determining for vague convergence.

Then we shall prove that

Proposition 0.1 *In the above bootstrap procedure, for all $H \in \mathcal{F}$,*

$$\begin{aligned}
&|EH(\xi_n) - EH(G_{mult,dep,samp})| \\
&\leq \frac{1}{\sqrt{n}} \sum_{i=1}^n |A(S_i) - 1| + \frac{\sqrt{n}}{\kappa^2} \sum_{i=1}^n \left| \sum_{a=1}^n \sum_{b=1}^n \left\{ \sum_{v \in S_a \cap S_b \cap S_i} \left(\frac{1}{\gamma_v}\right)^3 - \frac{1}{n} A(S_i) \sum_{v \in S_a \cap S_b} \left(\frac{1}{\gamma_v}\right)^2 \right\} \right| \\
&\quad + \frac{1}{n^{\frac{3}{2}} \kappa^2} \sum_{i=1}^n \sum_{j=1}^n \Big| nB(S_i \cap S_j) - A(S_i)A(S_j) \Big| \sum_{k=1}^n A(S_k).
\end{aligned}$$

Before proving Proposition 0.1 we derive the covariance structure for our bootstrap procedure.

Lemma 0.2

For our bootstrap procedure,

$$\mathrm{Cov}(\langle G_{mult,dep,samp}, f\rangle, \langle G_{mult,dep,samp}, g\rangle) = \frac{1}{n\kappa} \sum_{i=1}^n \sum_{j=1}^n \Big(nB(S_i \cap S_j) - A(S_i)A(S_j) \Big) f(x_i) g(x_j).$$

Proof First note that $\mathrm{Cov}(w_i - 1, w_j - 1) = \mathrm{Cov}(w_i, w_j)$ by linearity. To calculate this covariance, we view the multinomial vector $\mathbf{k} = (k_1, \ldots, k_n)$ as resulting from κ independent ball tosses into n urns, where each urn has probability $\frac{1}{n}$ of being hit. Thus, writing

$$T(i) = j \quad \text{if ball } i \text{ lands in urn } j,$$

the collection $((T(i) = j)_{i=1,\ldots,\kappa})$ are independent Bernoulli $\frac{1}{n}$-variables, and $k_j = \sum_{i=1}^{\kappa} \mathbf{1}(T(i) = j)$. Here $\mathbf{1}(\cdot)$ is the indicator function which equals 1 if the event in its argument is true, and 0 otherwise. Hence

$$Ek_i = \frac{\kappa}{n}, \quad \mathrm{Var}(k_i) = \frac{\kappa}{n}\left(1 - \frac{1}{n}\right) \quad \text{and} \quad \mathrm{Cov}(k_i, k_j) = -\frac{\kappa}{n^2}, \quad i \neq j.$$

We use these results to calculate the covariance as follows. First note that

$$Ew_j = \frac{n}{\kappa} \sum_{i \in S_j} \frac{1}{\gamma_i} Ek_i = \frac{n}{\kappa} \sum_{i \in S_j} \frac{\kappa}{\gamma_i n} = \sum_{i \in S_j} \frac{1}{\gamma_i} = A(S_j).$$

Also

$$\begin{aligned}
\mathrm{Var}(w_j) &= \frac{n^2}{\kappa^2} \sum_{i \in S_j} \frac{1}{\gamma_i^2} \mathrm{Var}(k_i) + \frac{n^2}{\kappa^2} \sum_{i \in S_j} \sum_{\ell \neq i, \ell \in S_j} \frac{1}{\gamma_i \gamma_\ell} \mathrm{Cov}(k_i, k_\ell) \\
&= \frac{n^2}{\kappa^2} \sum_{i \in S_j} \frac{1}{\gamma_i^2} \frac{\kappa}{n}\left(1 - \frac{1}{n}\right) - \frac{n^2}{\kappa^2} \sum_{i \in S_j} \sum_{\ell \neq i, \ell \in S_j} \frac{1}{\gamma_i \gamma_\ell} \frac{\kappa}{n^2} \\
&= \frac{n-1}{\kappa} \sum_{i \in S_j} \frac{1}{\gamma_i^2} - \frac{1}{\kappa} \sum_{i \in S_j} \sum_{\ell \neq i, \ell \in S_j} \frac{1}{\gamma_i \gamma_\ell}
\end{aligned}$$

$$
\begin{aligned}
&= \frac{n}{\kappa}\sum_{i\in S_j}\frac{1}{\gamma_i^2} - \frac{1}{\kappa}\sum_{i\in S_j}\sum_{\ell\in S_j}\frac{1}{\gamma_i\gamma_\ell} \\
&= \frac{1}{\kappa}\Big(nB(S_j) - A(S_j)^2\Big).
\end{aligned}
$$

For the covariance, we have

$$
\begin{aligned}
\mathrm{Cov}(w_j, w_k) &= \frac{n^2}{\kappa^2}\mathrm{Cov}\left(\sum_{i\in S_j}\frac{k_i}{\gamma_i}, \sum_{\ell\in S_k}\frac{k_\ell}{\gamma_\ell}\right) \\
&= \frac{n^2}{\kappa^2}\mathrm{Cov}\left(\sum_{i\in S_j\cap S_k}\frac{k_i}{\gamma_i} + \sum_{i\in\setminus S_k}\frac{k_i}{\gamma_i}, \sum_{\ell\in S_j\cap S_k}\frac{k_\ell}{\gamma_\ell} + \sum_{\ell\in\setminus S_k}\frac{k_\ell}{\gamma_\ell}\right) \\
&= \frac{n^2}{\kappa^2}\Bigg\{\mathrm{Var}\left(\sum_{i\in S_j\cap S_k}\frac{k_i}{\gamma_i}\right) + \mathrm{Cov}\left(\sum_{i\in\setminus S_k}\frac{k_i}{\gamma_i}, \sum_{\ell\in S_j\cap S_k}\frac{k_\ell}{\gamma_\ell}\right) \\
&\quad + \mathrm{Cov}\left(\sum_{i\in S_j\cap S_k}\frac{k_i}{\gamma_i}, \sum_{\ell\in\setminus S_k}\frac{k_\ell}{\gamma_\ell}\right) + \mathrm{Cov}\left(\sum_{i\in\setminus S_k}\frac{k_i}{\gamma_i}, \sum_{\ell\in\setminus S_k}\frac{k_\ell}{\gamma_\ell}\right)\Bigg\}.
\end{aligned}
$$

Now, by a similar argument used to calculate $\mathrm{Var}(w_i)$, for any subset $U \subset \{i, \ldots, n\}$,

$$
\mathrm{Var}\left(\sum_{i\in U}\frac{k_i}{\gamma_i}\right) = \frac{\kappa}{n^2}\left(n\sum_{i\in U}\frac{1}{\gamma_i^2} - A(U)^2\right).
$$

Moreover, for disjoint U and V,

$$
\mathrm{Cov}\Big(\sum_{i\in U}\frac{k_i}{\gamma_i}, \sum_{\ell\in V}\frac{k_\ell}{\gamma_\ell}\Big) = -\sum_{i\in U}\sum_{\ell\in V}\frac{1}{\gamma_i\gamma_l}\frac{\kappa}{n^2} = -\frac{\kappa}{n^2}A(U\cap V).
$$

So

$$
\begin{aligned}
\mathrm{Cov}(w_j, w_k) &= \frac{1}{\kappa}\Big\{n\sum_{i\in S_j\cap S_k}\frac{1}{\gamma_i^2} - (A(S_j\cap S_k))^2 - A(S_j\setminus S_k)A(S_j\cap S_k) \\
&\quad - A(S_j\cap S_k)A(S_k\setminus S_j) - A(S_j\setminus S_k)A(S_k\setminus S_j)\Big\} \\
&= \frac{1}{\kappa}\Big\{n\sum_{i\in S_j\cap S_k}\frac{1}{\gamma_i^2} - (A(S_j\cap S_k) + A(S_j\setminus S_k))(A(S_j\cap S_k)A(S_k\setminus S_j))\Big\} \\
&= \frac{1}{\kappa}\Big\{n\sum_{i\in S_j\cap S_k}\frac{1}{\gamma_i^2} - A(S_j)A(S_k)\Big\} \\
&= \frac{1}{\kappa}\Big\{nB(S_j\cap S_k) - A(S_j)A(S_k)\Big\}. \qquad (4)
\end{aligned}
$$

Hence we obtain that

$$
\begin{aligned}
\mathrm{Cov}(\langle G_{mult,dep,samp}, f\rangle, \langle G_{mult,dep,samp}, g\rangle) &= \frac{1}{n\kappa}\sum_{i=1}^{n}\Big(nB(S_i) - A(S_i)^2\Big)f(x_i)g(x_i) \\
&+ \frac{1}{n\kappa}\sum_{i=1}^{n}\sum_{j\neq i}\Big(nB(S_i\cap S_j) - A(S_i)A(S_j)\Big)f(x_i)g(x_j).
\end{aligned}
$$

Combining the case that $j = i$ and that $j \neq i$ finishes the proof. Q.E.D.

The proof of Proposition 0.1 is based on Stein's method for empirical measures, see [11]. We equip the space $M^f(I\!R)$ of real-valued bounded Radon measures on $I\!R$ with the topology of vague convergence, as follows. Let $C_c(I\!R)$ be the space of real-valued continuous functions on $I\!R$ with support contained in a compact set. Let $(\nu_n)_n$ be a family of measures in $M^f(I\!R)$, and let ν be a measure in $M^f(I\!R)$. We say that ν_n converges vaguely to ν, in short, $\nu_n \stackrel{v}{\Rightarrow} \nu$, if and only if for all functions $f \in C_c(I\!R)$ we have $\langle \nu_n, f\rangle \to \langle \nu, f\rangle$ as $n \to \infty$. Here we use the notation

$$\langle \nu, f\rangle = \int f d\nu.$$

To describe a Gaussian random measure we assume that $b : C_b^\infty(I\!R) \times C_b^\infty(I\!R) \to I\!R$ is a quadratic form such that, for any $m \in \mathbf{N}$ and for all $\phi_1, \dots, \phi_m \in C_b^\infty(I\!R)$,

$$B = B(\phi_1, \dots, \phi_m) = (b(\phi_i, \phi_j))_{i,j=1,\dots,m}$$

is a symmetric, positive definite matrix. With the abbreviation

$$\underline{\langle \nu, \phi\rangle} = (\langle \nu, \phi_1\rangle, \dots, \langle \nu, \phi_m\rangle),$$

we define the generator associated with the operator b by

$$\mathcal{A}F(\nu) = -\sum_{j=1}^m f_{(j)}(\underline{\langle \nu, \phi\rangle})\langle \nu, \phi_j\rangle + \sum_{j,k=1}^m f_{(j,k)}(\underline{\langle \nu, \phi\rangle}) b(\phi_j, \phi_k).$$

Let ζ be a random measure taking values in the space of finite signed measures $M^f(I\!R)$ almost surely such that, for all $m \in \mathbf{N}$, and for all $\phi_1, \dots, \phi_m \in C_b^\infty(I\!R)$,

$$\mathcal{L}(\langle \zeta, \phi_1\rangle, \dots, \langle \zeta, \phi_m\rangle) = \mathcal{MVN}_m(0, B),$$

where $\mathcal{MVN}_m(0, B)$ denotes the multivariate normal law with mean vector 0 and covariance matrix B. Then ζ is a Gaussian random measure. Moreover, for $H \in \mathcal{F}$ of the form

$$H(\nu) = h(\langle \nu, \psi_1\rangle, \dots, \langle \nu, \psi_m\rangle), \tag{5}$$

the so-called *Stein equation* corresponding to the Gaussian random measure ζ is

$$h(\underline{\langle \nu, \phi\rangle}) - Eh(\underline{\langle \zeta, \phi\rangle}) = -\sum_{j=1}^m f_{(j)}(\underline{\langle \nu, \phi\rangle})\langle \nu, \phi_j\rangle + \sum_{j,k=1}^m f_{(j,k)}(\underline{\langle \nu, \phi\rangle}) b(\phi_j, \phi_k). \tag{6}$$

The equation can be solved using a semigroup technique as in [12] (see also [13]); for each $H \in \mathcal{F}$ has the form (5), there is a function $F \in \mathcal{F}$, and there is a function $f \in C_b^\infty(I\!R^m)$ such that $F(\nu) = f(\langle \nu, \psi_1\rangle, \dots, \langle \nu, \psi_m\rangle)$, and $\|f^{(k)}\| \leq \|h^{(k)}\|, k \in \mathbf{N}$.

To prove a Gaussian approximation, we may employ the following result.

Proposition 0.3 *Let $(\eta_n)_{n \in \{1,2,\dots\}}$ be a family of random measures taking values in $M^f(I\!R)$ almost surely. Let ζ be a random measure taking values in $M^f(I\!R)$ almost surely such that, for all $m \in \mathbf{N}, \phi_1, \dots, \phi_m \in C_b^\infty(I\!R)$,*

$$\mathcal{L}(\langle \zeta, \phi_1\rangle, \dots, \langle \zeta, \phi_m\rangle) = \mathcal{MVN}_m(0, B).$$

Let $\mathcal{A}$ be the generator associated with B. Let H be of the form (5) and let F be the solution of the Stein equation (6). Then

$$|EH(\eta_n) - EH(\zeta)| = |E\mathcal{A}F(\eta_n)|.$$

Now we have the necessary ingredients assembled to prove Proposition 0.1.

Proof of Proposition 0.1.

Let H be of the form (5) and let F be the solution of the Stein equation (6); write F in the form (1). Then as suggested in Proposition 0.3 we bound

$$E\mathcal{A}F(\xi_n) = -E\sum_{j=1}^{m} f_{(j)}(\underline{\langle \xi_n, \phi\rangle})\langle \xi_n, \phi_j\rangle + E\sum_{j,k=1}^{m} f_{(j,k)}(\underline{\langle \xi_n, \phi\rangle})b(\phi_j, \phi_k),$$

where the covariance operator is as in Lemma ,

$$b(\phi_j, \phi_k) = \frac{1}{n\kappa}\sum_{i=1}^{n}\sum_{j=1}^{n}\Big(nB(S_i \cap S_j) - A(S_i)A(S_j)\Big)\phi_j(x_i)\phi_k(x_j).$$

We start with the term $E\sum_{j=1}^{m} f_{(j)}(\underline{\langle \xi_n, \phi\rangle})\langle \xi_n, \phi_j\rangle$; writing out ξ_n we obtain

$$E\sum_{j=1}^{m} f_{(j)}(\underline{\langle \xi_n, \phi\rangle})\langle \xi_n, \phi_j\rangle = \frac{1}{\sqrt{n}}\sum_{i=1}^{n}\sum_{j=1}^{m} Ef_{(j)}(\underline{\langle \xi_n, \phi\rangle})\phi_j(x_i)(w_i - 1).$$

Recall that $Ew_j = A(S_j)$; we expand the right-hand side as

$$\begin{aligned}
&\frac{1}{\sqrt{n}}\sum_{i=1}^{n}\sum_{j=1}^{m} Ef_{(j)}(\underline{\langle \xi_n, \phi\rangle})\phi_j(x_i)(w_i - 1)\\
&= \quad \frac{1}{\sqrt{n}}\sum_{i=1}^{n}\sum_{j=1}^{m} Ef_{(j)}(\underline{\langle \xi_n, \phi\rangle})\phi_j(x_i)(w_i - A(S_i)) + R_1,
\end{aligned}$$

where

$$R_1 = \frac{1}{\sqrt{n}}\sum_{i=1}^{n}\sum_{j=1}^{m} Ef_{(j)}(\underline{\langle \xi_n, \phi\rangle})\phi_j(x_i)(A(S_i) - 1).$$

Using the definition of $\mathcal{F}$ we can bound

$$|R_1| \le \frac{1}{\sqrt{n}}\sum_{i=1}^{n}|A(S_i) - 1|.$$

With $T(i) = j$ if ball i lands in urn j in our sub-sampling procedure we expand

$$w_i = \frac{n}{\kappa}\sum_{t\sim i}\sum_{l=1}^{\kappa}\mathbf{1}(T(l) = t)\frac{1}{\gamma_t}.$$

This gives also that

$$A(S_i) = Ew_i = \frac{n}{\kappa}\sum_{t\sim i}\sum_{l=1}^{\kappa}\frac{1}{n}\frac{1}{\gamma_t}.$$

Now we expand further

$$\begin{aligned}
&\frac{1}{\sqrt{n}}\sum_{i=1}^{n}\sum_{j=1}^{m} Ef_{(j)}(\underline{\langle\xi_n,\phi\rangle})\phi_j(x_i)(w_i - A(S_i)) \\
&= \frac{n}{\kappa}\frac{1}{\sqrt{n}}\sum_{i=1}^{n}\sum_{j=1}^{m}\sum_{t\sim i}\sum_{l=1}^{\kappa} Ef_{(j)}(\underline{\langle\xi_n,\phi\rangle})\phi_j(x_i)\left(\mathbf{1}(T(l)=t)\frac{1}{\gamma_t} - A(S_i)\right) \\
&= \frac{\sqrt{n}}{\kappa}\sum_{i=1}^{n}\sum_{j=1}^{m}\sum_{t\sim i}\sum_{l=1}^{\kappa} Ef_{(j)}(\underline{\langle\xi_n,\phi\rangle})\phi_j(x_i)\left(\mathbf{1}(T(l)=t) - \frac{1}{n}\right)\frac{1}{\gamma_t}.
\end{aligned}$$

Now we shall exploit that $\mathbf{1}(T(l)=t)$ and w_j are typically only weakly dependent. To this purpose let $w_j^{(l)}$ denote the weights re-calculated without ball l; then

$$w_j - w_j^{(l)} = \frac{n}{\kappa}\sum_{v\in S_j}\frac{1}{\gamma_v}\mathbf{1}(T(l)=v)$$

and similarly we define $\xi_n^{(l)}$ so that

$$\langle \xi_n - \xi_n^{(l)}, \phi\rangle = \frac{1}{\sqrt{n}}\sum_{i=1}^{n}\phi(x_i)\left(w_i - w_i^{(l)}\right).$$

Note that this difference depends only on the random quantity $T(l)$ and not on any of the other balls. With Taylor expansion,

$$\begin{aligned}
&\frac{\sqrt{n}}{\kappa}\sum_{i=1}^{n}\sum_{j=1}^{m}\sum_{t\sim i}\sum_{l=1}^{\kappa} Ef_{(j)}(\underline{\langle\xi_n,\phi\rangle})\phi_j(x_i)\left(\mathbf{1}(T(l)=t) - \frac{1}{n}\right)\frac{1}{\gamma_t} \\
&= \frac{\sqrt{n}}{\kappa}\sum_{i=1}^{n}\sum_{j=1}^{m}\sum_{t\sim i}\sum_{l=1}^{\kappa} Ef_{(j)}(\underline{\langle\xi_n^{(l)},\phi\rangle})\phi_j(x_i)\left(\mathbf{1}(T(l)=t) - \frac{1}{n}\right)\frac{1}{\gamma_t} \\
&\quad + \frac{\sqrt{n}}{\kappa}\sum_{i=1}^{n}\sum_{j=1}^{m}\sum_{t\sim i}\sum_{l=1}^{\kappa}\sum_{k=1}^{m} Ef_{(j,k)}(\underline{\langle\xi_n^{(l)},\phi\rangle})\langle\xi_n - \xi_n^{(l)},\phi_k\rangle\phi_j(x_i)\left(\mathbf{1}(T(l)=t) - \frac{1}{n}\right)\frac{1}{\gamma_t} + R_2,
\end{aligned}$$

where, for some $0 < \theta < 1$,

$$\begin{aligned}
R_2 &= \frac{\sqrt{n}}{\kappa}\sum_{i=1}^{n}\sum_{j=1}^{m}\sum_{t\sim i}\sum_{l=1}^{\kappa}\sum_{k=1}^{m}\sum_{w=1}^{m} Ef_{(j,k,w)}(\underline{\langle\theta\xi_n + (1-\theta)\xi^{(l)},\phi\rangle})\langle\xi_n - \xi_n^{(l)},\phi_k\rangle \\
&\quad \times\langle\xi_n - \xi_n^{(l)},\phi_w\rangle\phi_j(x_i)\left(\mathbf{1}(T(l)=t) - \frac{1}{n}\right)\frac{1}{\gamma_t}.
\end{aligned}$$

We shall bound R_2 later. By independence we have that

$$Ef_{(j)}(\underline{\langle\xi^{(l)},\phi\rangle})\phi_j(x_i)\left(\mathbf{1}(T(l)=t) - \frac{1}{n}\right)\frac{1}{\gamma_t} = 0$$

and

$$\begin{aligned}
&\frac{\sqrt{n}}{\kappa}\sum_{i=1}^{n}\sum_{j=1}^{m}\sum_{t\sim i}\sum_{l=1}^{\kappa} Ef_{(j)}(\underline{\langle\xi,\phi\rangle})\phi_j(x_i)\left(\mathbf{1}(T(l)=t) - \frac{1}{n}\right)\frac{1}{\gamma_t} \\
&= \frac{\sqrt{n}}{\kappa}\sum_{i=1}^{n}\sum_{j=1}^{m}\sum_{t\sim i}\sum_{l=1}^{\kappa}\sum_{k=1}^{m} Ef_{(j,k)}(\underline{\langle\xi_n^{(l)},\phi\rangle})E\langle\xi_n - \xi_n^{(l)},\phi_k\rangle\phi_j(x_i)\left(\mathbf{1}(T(l)=t) - \frac{1}{n}\right)\frac{1}{\gamma_t} + R_2.
\end{aligned}$$

Next we note that

$$\begin{aligned}
&\frac{\sqrt{n}}{\kappa}\sum_{i=1}^{n}\sum_{t\sim i}E\langle\xi_n-\xi_n^{(l)},\phi_k\rangle\phi_j(x_i)\left(\mathbf{1}(T(l)=t)-\frac{1}{n}\right)\frac{1}{\gamma_t}\\
&= \frac{1}{\kappa}\sum_{i=1}^{n}\sum_{t\sim i}\sum_{f=1}^{n}\phi_k(x_f)\phi_j(x_i)E\left(w_f-w_f^{(l)}\right)\left(\mathbf{1}(T(l)=t)-\frac{1}{n}\right)\frac{1}{\gamma_t}\\
&= \frac{n}{\kappa^2}\sum_{i=1}^{n}\sum_{t\sim i}\sum_{f=1}^{n}\phi_k(x_f)\phi_j(x_i)\sum_{v\in S_f}\frac{1}{\gamma_v}\frac{1}{\gamma_t}E\mathbf{1}(T(l)=v)\left(\mathbf{1}(T(l)=t)-\frac{1}{n}\right)\\
&= \frac{n}{\kappa^2}\sum_{i=1}^{n}\sum_{t\sim i}\sum_{f=1}^{n}\phi_k(x_f)\phi_j(x_i)\sum_{v\in S_f}\frac{1}{\gamma_v}\frac{1}{\gamma_t}\left(-\frac{1}{n^2}+\mathbf{1}(v=t)\frac{1}{n}\right)\\
&= \frac{1}{n\kappa^2}\sum_{i=1}^{n}\sum_{f=1}^{n}\phi_k(x_f)\phi_j(x_i)(nB(S_f\cap S_v)-A(S_f)A(S_g))\\
&= \frac{1}{\kappa}b(\phi_j,\phi_k).
\end{aligned}$$

Hence

$$\begin{aligned}
&\frac{\sqrt{n}}{\kappa}\sum_{i=1}^{n}\sum_{j=1}^{m}\sum_{t\sim i}\sum_{l=1}^{\kappa}\sum_{k=1}^{m}Ef_{(j,k)}(\underline{\langle\xi_n^{(l)},\phi\rangle})\\
&\qquad\times E\langle\xi_n-\xi_n^{(l)},\phi_k\rangle\phi_j(x_i)\left(\mathbf{1}(T(l)=t)-\frac{1}{n}\right)\frac{1}{\gamma_t}\\
&= \frac{1}{\kappa}\sum_{j=1}^{m}\sum_{k=1}^{m}b(\phi_j,\phi_k)\sum_{l=1}^{\kappa}Ef_{(j,k)}(\underline{\langle\xi_n^{(l)},\phi\rangle})\\
&= \sum_{j=1}^{m}\sum_{k=1}^{m}b(\phi_j,\phi_k)Ef_{(j,k)}(\underline{\langle\xi_n,\phi\rangle})+R_3,
\end{aligned}$$

where

$$R_3=\frac{1}{\kappa}\sum_{j=1}^{m}\sum_{k=1}^{m}b(\phi_j,\phi_k)\sum_{l=1}^{\kappa}E\left\{f_{(j,k)}(\underline{\langle\xi_n^{(l)},\phi\rangle})-f_{(j,k)}(\underline{\langle\xi_n,\phi\rangle})\right\}.$$

Assembling the argument we have shown so far that

$$E\mathcal{A}F(\xi_n)=-E\sum_{j=1}^{m}f_{(j)}(\underline{\langle\xi,\phi\rangle})\langle\xi,\phi_j\rangle+E\sum_{j,k=1}^{m}f_{(j,k)}(\underline{\langle\xi,\phi\rangle})b(\phi_j,\phi_k)=R_1+R_2+R_3,$$

where $|R_1|\le\frac{1}{\sqrt{n}}\sum_{i=1}^{n}|A(S_i)-1|$. It remains to bound R_2 and R_3. For R_2,

$$\begin{aligned}
|R_2| \;\le\; &\frac{\sqrt{n}}{\kappa}\sum_{i=1}^{n}\sum_{j=1}^{m}\sum_{l=1}^{\kappa}\sum_{k=1}^{m}\sum_{w=1}^{m}||f_{(j,k,w)}||\\
&\times E\left|\sum_{t\sim i}\frac{1}{\gamma_t}\langle\xi_n-\xi_n^{(l)},\phi_k\rangle\langle\xi_n-\xi_n^{(l)},\phi_w\rangle\left(\mathbf{1}(T(l)=t)-\frac{1}{n}\right)\right|
\end{aligned}$$

$$
\begin{aligned}
&= \frac{\sqrt{n}}{n\kappa} \times \frac{n^2}{\kappa^2} \sum_{i=1}^n \sum_{j=1}^m \sum_{t \sim i} \frac{1}{\gamma_t} \sum_{l=1}^{\kappa} \sum_{k=1}^m \sum_{w=1}^m ||f_{(j,k,w)}|| \\
&\quad \times E \left| \sum_{a=1}^n \sum_{b=1}^n \phi_k(x_a)\phi_w(x_b) \sum_{v \in S_a} \frac{1}{\gamma_v} \mathbf{1}(T(l)=v) \sum_{x \in S_b} \frac{1}{\gamma_x} \mathbf{1}(T(l)=x) \left(\mathbf{1}(T(l)=t) - \frac{1}{n} \right) \right|.
\end{aligned}
$$

Now, ball l lands in exactly one bin, and so $\mathbf{1}(T(l)=v)\mathbf{1}(T(l)=x) = 0$ unless $x = v$. Hence

$$
\begin{aligned}
& E \left| \sum_{t \sim i} \frac{1}{\gamma_t} \sum_{a=1}^n \sum_{b=1}^n \phi_k(x_a)\phi_w(x_b) \sum_{v \in S_a} \frac{1}{\gamma_v} \mathbf{1}(T(l)=v) \sum_{x \in S_b} \frac{1}{\gamma_x} \mathbf{1}(T(l)=x) \left(\mathbf{1}(T(l)=t) - \frac{1}{n} \right) \right| \\
&= E \left| \sum_{t \sim i} \frac{1}{\gamma_t} \sum_{a=1}^n \sum_{b=1}^n \phi_k(x_a)\phi_w(x_b) \sum_{v \in S_a \cap S_b} \left(\frac{1}{\gamma_v} \right)^2 \mathbf{1}(T(l)=v) \left(\mathbf{1}(T(l)=t) - \frac{1}{n} \right) \right| \\
&= E \Bigg| \sum_{t \sim i} \frac{1}{\gamma_t} \sum_{a=1}^n \sum_{b=1}^n \phi_k(x_a)\phi_w(x_b) \Bigg\{ \sum_{v \in S_a \cap S_b \cap S_t} \left(\frac{1}{\gamma_v} \right)^2 \mathbf{1}(T(l)=v) \\
&\quad - \frac{1}{n} \sum_{v \in S_a \cap S_b} \left(\frac{1}{\gamma_v} \right)^2 \mathbf{1}(T(l)=t) \Bigg\} \Bigg| \\
&\le \frac{1}{n} \left| \sum_{a=1}^n \sum_{b=1}^n \left\{ \sum_{v \in S_a \cap S_b \cap S_i} \left(\frac{1}{\gamma_v} \right)^3 - A(S_i) \frac{1}{n} \sum_{v \in S_a \cap S_b} \left(\frac{1}{\gamma_v} \right)^2 \right\} \right|.
\end{aligned}
$$

For R_2 we hence obtain the bound

$$
\begin{aligned}
|R_2| &\le \frac{\sqrt{n}}{n} \times \frac{n^2}{\kappa^2} \times \frac{1}{n} \sum_{i=1}^n \left| \sum_{a=1}^n \sum_{b=1}^n \left\{ \sum_{v \in S_a \cap S_b \cap S_i} \left(\frac{1}{\gamma_v} \right)^3 - \frac{1}{n} A(S_i) \sum_{v \in S_a \cap S_b} \left(\frac{1}{\gamma_v} \right)^2 \right\} \right| \\
&= \frac{\sqrt{n}}{\kappa^2} \sum_{i=1}^n \left| \sum_{a=1}^n \sum_{b=1}^n \left\{ \sum_{v \in S_a \cap S_b \cap S_i} \left(\frac{1}{\gamma_v} \right)^3 - \frac{1}{n} A(S_i) \sum_{v \in S_a \cap S_b} \left(\frac{1}{\gamma_v} \right)^2 \right\} \right|.
\end{aligned}
$$

For R_3, using Taylor expansion and the bounds on ϕ_w as well as on the third partial derivatives of f,

$$
\begin{aligned}
|R_3| &\le \frac{1}{\kappa} \sum_{j=1}^m \sum_{k=1}^m |b(\phi_j, \phi_k)| \sum_{l=1}^{\kappa} \sum_{w=1}^m ||f_{(j,k,w)}|| E |\langle \xi_n^{(l)} - \xi_n, \phi_w \rangle| \\
&\le \frac{1}{\sqrt{n}\kappa} \sum_{i=1}^n \sum_{j=1}^m \sum_{k=1}^m |b(\phi_j, \phi_k)| \sum_{l=1}^{\kappa} \sum_{w=1}^m ||f_{(j,k,w)}|| |\phi_w(x_i)| E|w_i - w_i^{(l)}| \\
&\le \frac{\sqrt{n}}{\kappa^2} \sum_{i=1}^n \sum_{j=1}^m \sum_{k=1}^m |b(\phi_j, \phi_k)| \sum_{l=1}^{\kappa} \sum_{w=1}^m ||f_{(j,k,w)}|| |\phi_w(x_i)| \sum_{v \in S_i} \frac{1}{\gamma_v} E \mathbf{1}(T(l)=v) \\
&= \frac{1}{\sqrt{n}\kappa^2} \sum_{i=1}^n \sum_{j=1}^m \sum_{k=1}^m |b(\phi_j, \phi_k)| \sum_{l=1}^{\kappa} \sum_{w=1}^m ||f_{(j,k,w)}|| |\phi_w(x_i)| \sum_{v \in S_i} \frac{1}{\gamma_v} \\
&\le \frac{1}{\sqrt{n}\kappa} \sum_{i=1}^n \sum_{j=1}^m \sum_{k=1}^m |b(\phi_j, \phi_k)| \sum_{w=1}^m ||f_{(j,k,w)}|| A(S_i).
\end{aligned}
$$

We bound

$$|b(\phi_j, \phi_k)| \leq \frac{1}{n\kappa} \sum_{i=1}^{n} \sum_{j=1}^{n} \Big| nB(S_i \cap S_j) - A(S_i)A(S_j) \Big|.$$

This bound gives that

$$|R_3| \leq \frac{1}{n^{\frac{3}{2}}\kappa^2} \sum_{i=1}^{n} \sum_{j=1}^{n} \Big| nB(S_i \cap S_j) - A(S_i)A(S_j) \Big| \sum_{k=1}^{n} A(S_k).$$

Collecting the bounds yields the assertion. Q.E.D.

References

[1] Erdös, P. & Rényi, A. On the evolution of random graphs. *Publication of the Mathematical Institute of the Hungarian Academy of Sciences* **5**, 17–61 (1960).

[2] Bollobás, B. A probabilistic proof of an asymptotic formula for the number of labelled regular graphs. *European Journal of Combinatorics* **1**, 311–316 (1980).

[3] Molloy, M. & Reed, B. A. A critical point for random graphs with a given degree sequence. *Random Structures and Algorithms* **6**, 161–180 (1995).

[4] Newman, M. E., Strogatz, S. H. & Watts, D. J. Random graphs with arbitrary degree distributions and their applications. *Physical Review E* **64**, 026118 (2001).

[5] Gilbert, E. N. Random plane networks. *Journal of the Society for Industrial & Applied Mathematics* **9**, 533–543 (1961).

[6] Penrose, M. *Random geometric graphs* (Oxford University Press Oxford, 2003).

[7] Przulj, N., Kuchaiev, O., Stevanovic, A. & Hayes, W. Geometric evolutionary dynamics of protein interaction networks. In *Pacific Symposium on Biocomputing*, vol. 2009, 178–189 (World Scientific, 2010).

[8] Chung, F. & Lu, L. The average distances in random graphs with given expected degrees. *Proceedings of the National Academy of Sciences of the United States of America* **99**, 15879–15882 (2002).

[9] Middendorf, M. *et al.* Inferring network mechanisms: The drosophila melanogaster protein interaction network. *Proceedings of the National Academy of Sciences of the United States of America* **102**, 3192–3197 (2005).

[10] Gibson, T. A. & Goldberg, D. S. Improving evolutionary models of protein interaction networks. *Bioinformatics* **27**, 376–382 (2011).

[11] Reinert, G. A weak law of large numbers for empirical measures via stein's method. *The Annals of Probability* 334–354 (1995).

[12] Barbour, A. D. Stein's method and poisson process convergence. *Journal of Applied Probability* 175–184 (1988).

[13] Goldstein, L. & Rinott, Y. Multivariate normal approximations by stein's method and size bias couplings. *Journal of Applied Probability* 1–17 (1996).
